# Structural and Biochemical Characterization of the Cop9 Signalosome CSN5/CSN6 Heterodimer

**DOI:** 10.1371/journal.pone.0105688

**Published:** 2014-08-21

**Authors:** Melissa Birol, Radoslav Ivanov Enchev, André Padilla, Florian Stengel, Ruedi Aebersold, Stéphane Betzi, Yinshan Yang, François Hoh, Matthias Peter, Christian Dumas, Aude Echalier

**Affiliations:** 1 Centre de Biochimie Structurale, Unité Mixte de Recherche (UMR) 5048, Centre National de Recherche Scientifique (CNRS), Université Montpellier 1 (UM1), Université Montpellier 2 (UM2), Montpellier, France; 2 Institut national de la santé et de la recherche médicale (INSERM) U1054, Paris, France; 3 ETH Zurich, Department of Biology, Institute of Biochemistry, Zurich, Switzerland; 4 ETH Zurich, Department of Biology, Institute of Molecular Systems Biology, Zurich, Switzerland; 5 Faculty of Science, University of Zurich, Zurich, Switzerland; 6 Centre de Recherche en Cancérologie de Marseille, Centre de Biochimie Structurale, Unité Mixte de Recherche (UMR) 7258, Institut national de la santé et de la recherche médicale (INSERM) U1068, Institut Paoli-Calmettes, Aix-Marseille Université UM105, Marseille, France; University of Copenhagen, Denmark

## Abstract

The Cop9 signalosome complex (CSN) regulates the functional cycle of the major E3 ubiquitin ligase family, the cullin RING E3 ubiquitin ligases (CRLs). Activated CRLs are covalently modified by the ubiquitin-like protein Nedd8 (neural precursor cell expressed developmentally down-regulated protein 8). CSN serves an essential role in myriad cellular processes by reversing this modification through the isopeptidase activity of its CSN5 subunit. CSN5 alone is inactive due to an auto-inhibited conformation of its catalytic domain. Here we report the molecular basis of CSN5 catalytic domain activation and unravel a molecular hierarchy in CSN deneddylation activity. The association of CSN5 and CSN6 MPN (for Mpr1/Pad1 N-terminal) domains activates its isopeptidase activity. The CSN5/CSN6 module, however, is inefficient in CRL deneddylation, indicating a requirement of further elements in this reaction such as other CSN subunits. A hybrid molecular model of CSN5/CSN6 provides a structural framework to explain these functional observations. Docking this model into a published CSN electron density map and using distance constraints obtained from cross-linking coupled to mass-spectrometry, we find that the C-termini of the CSN subunits could form a helical bundle in the centre of the structure. They likely play a key scaffolding role in the spatial organization of CSN and precise positioning of the dimeric MPN catalytic core.

## Introduction

The ubiquitin-proteasome system is implicated in virtually all functions of eukaryotic living cells. The covalent attachment of ubiquitin molecules, ubiquitylation, requires the concerted intervention of the distinct E1, E2 and E3 enzymes. The most prominent E3 ubiquitin ligase family comprises the cullin RING E3 ubiquitin ligases (CRLs). CRLs are important regulators of cellular homeostasis, division, and responses to various cellular insults [1]. Their significance is further highlighted by the deregulation of some CRL elements in human diseases and, in particular, in cancers [2], [3].

CRLs are built around a cullin scaffold, which associates with a substrate-specific adaptor and a RING finger protein [4]. CRL activity is tightly regulated to ensure the timely and specific substrate ubiquitylation [1]. The covalent attachment of the ubiquitin-like (UBL) molecule Nedd8 (neural precursor cell expressed developmentally down-regulated protein 8) to cullins, termed neddylation, activates their ubiquitylation activity by stabilising an activated conformation of the RING subunit [5], [6].

The Cop9 signalosome (CSN) is an eight-subunit complex that deneddylates cullins [7]. Six of its subunits (CSN1 to CSN4, CSN7, CSN8) contain a PCI (for Proteasome Cop9 eIF3) domain and two (CSN5/6) contain an MPN (for Mpr1/Pad1 N-terminal) domain. The PCI subunits serve a scaffolding function and, especially through CSN2, help recruit the neddylated CRL substrates [8]. Structurally, the PCI-containing subunits arrange in a horseshoe-like shape, juxtaposed with the two MPN subunits. This architecture is strikingly similar to that of the 26S proteasome lid and the eukaryotic translation initiation factor eIF3 [8], [9], [10], [11], [12], [13].

Among the CSN MPN-containing subunits, CSN6 lacks a functional catalytic site and belongs to the MPN^−^ class [14], [15], [16]. In contrast, CSN5, also known as Jun-activatory binding protein 1 (Jab1) carries the catalytically competent MPN^+^/JAMM (Jab1-MPN-Mov34) motif [15], [17]. As defined in the AMSH (Associated Molecule with the SH3 domain of STAM)-like protein (AMSH-LP) [18], the MPN domain of CSN5 contains two insertions, namely Insertion-1 (Ins-1; residues 97–131 in human CSN5) and Insertion-2 (Ins-2; residues 197–219 in human CSN5). These regions usually contribute to the regulation of the isopeptidase activity. For AMSH-LP, the Ins-1 region plays a role in the binding and competent positioning of the distal ubiquitin [18]. CSN5 by itself is found in an inactive state and the transition from this stand-alone auto-inhibited to the active form probably requires conformational changes of the Ins-1 region [17], [19]. This is reminiscent of Rpn11 and BRCC36 (for BRCA1 (for Breast Cancer 1)/BRCA2 (for Breast Cancer 2)-Containing Complex subunit 36) found in the 26S proteasome lid and BRCC36-containing complexes, respectively, that are also inactive in the stand-alone form [20]. Recently, the crystal structure of Rpn11/Rpn8 MPN domains brought important insights into the dimerisation of an MPN^+^/JAMM enzyme with an MPN^−^ subunit [21], [22], pointing towards the mechanism underlying the catalytic activation of inactive MPN^+^/JAMM enzymes upon integration in higher order assemblies.

Although the structural and functional understanding of the CSN/CRL interplay has significantly grown over the recent years, important questions concerning the association of the MPN domains and the regulation of CSN activity remain. Here we report that CSN6 N-terminal domain containing its MPN domain can form a stable heterodimer with CSN5 catalytic domain *in vitro*. Biophysical and activity measurements indicate that CSN6 MPN domain stimulates CSN5's isopeptidase activity. Comparison with CSN on synthetic substrates reveals that both CSN and the dimeric MPN module display robust isopeptidase activity towards C-terminal Nedd8 derivatives, but that CSN is a more efficient deneddylase towards Cullin1-Nedd8 than the CSN5/CSN6 MPN complex. This suggests that the MPN^−^ subunit would contribute significantly to the catalytic activity of the human CSN, but efficient substrate recruitment would require additional elements such as other CSN subunits. Moreover, we provide a structural context for these observations, combining X-ray crystallography, NMR and cross-linking coupled to MS (CX-MS) derived data and flexible molecular docking to produce a molecular model of the CSN5/CSN6 MPN heterodimer and re-interpret the molecular model of CSN (8). This analysis suggests that a major part of the subunit-subunit interactions in CSN are mediated by a helical bundle, composed of the C-termini of CSN's subunits, further highlighting its structural similarity to the 26S proteasome lid [23]. Our work indicates that, in addition to the importance of their MPN domains for catalysis, the C-terminal regions of CSN5 and of CSN6 contribute to their anchoring and precise positioning in the CSN assembly.

## Experimental Procedures

### Protein production

**Figure 1:**
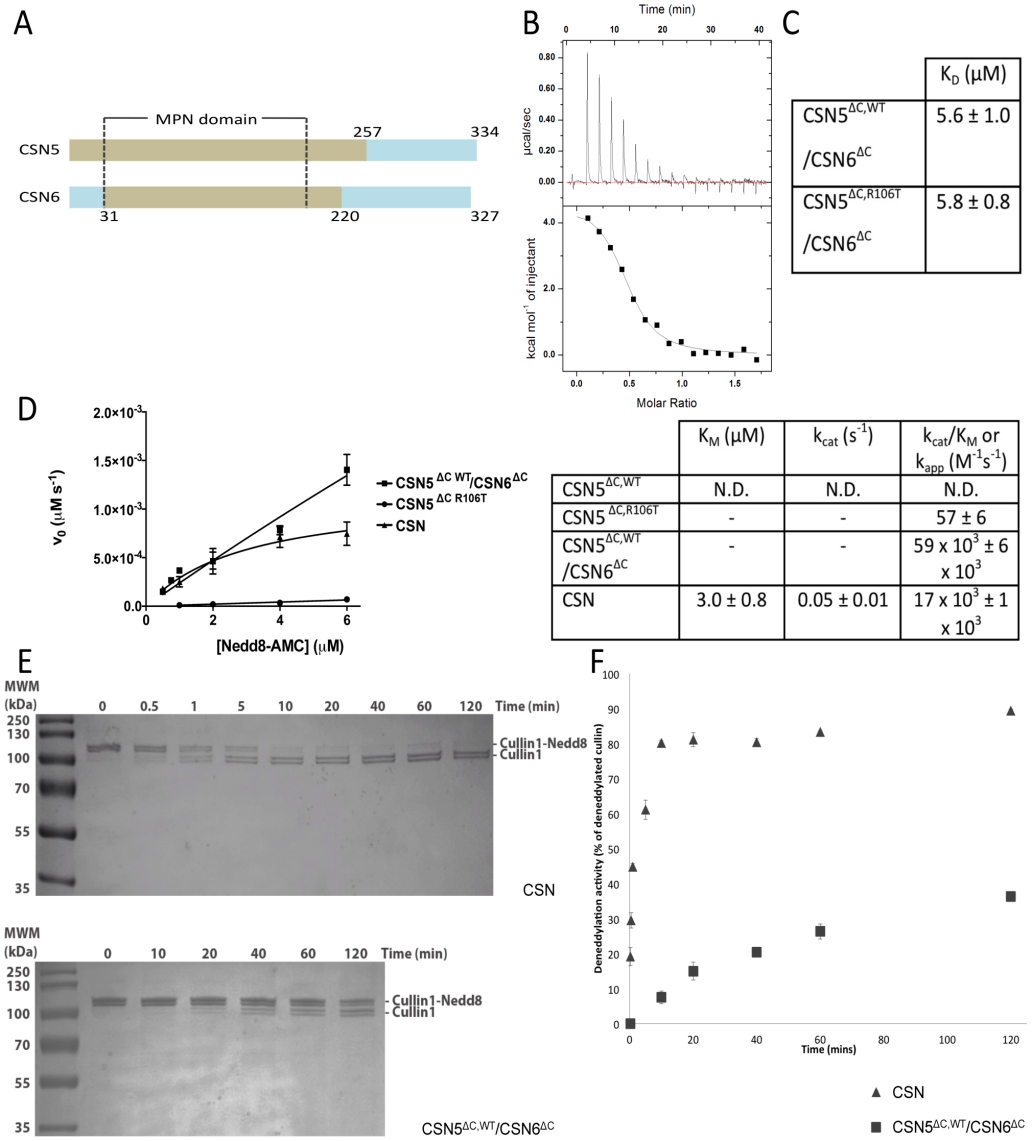
CSN6^ΔC^ activates CSN5^ΔC^. (**A**) **Studied protein fragments (brown)**. The MPN domain boundaries are indicated with dotted lines. (**B**) **Binding of CSN5^ΔC^ and CSN6^ΔC^ measured by ITC.** Titration experiments were carried out by injecting concentrated CSN6^ΔC^ solution into 100 µM CSN5 variants. (**C**) **K_D_ values of CSN5^ΔC^/CSN6^ΔC^ pairs obtained from ITC data.** (**D**) **Activity towards Nedd8-AMC.** Nedd8-AMC hydrolysis rate plot versus substrate concentration (200 nM CSN5^ΔC^ alone; 4 nM CSN5^ΔC^/CSN6^ΔC^ complex; 23 nM CSN), with data fitted using the Michaelis-Menten equation. Below: Associated kinetic parameters of the Nedd8-AMC cleavage derived from the left plot. In the CSN5^ΔC,R106T^ and CSN5^ΔC,WT^/CSN6^ΔC^ experiments, substrate saturation conditions were not reached and k_app_ values were extracted from linear fitting of the k_obs_ versus substrate concentration data. (**E**) **Time-course deneddylation of Cullin1-Nedd8/Rbx1 (1 µM) by the CSN (4 nM; Top) or CSN5^ΔC,WT^/CSN6 (4 nM; Bottom).** Samples from each time point were analysed on Coomassie stained 15% Tris-tricine SDS-PAGE. Quantification of the cullin deneddylation was carried out. The bands corresponding to Cullin1-Nedd8 and to Cullin1 show doublet that arise from Cullin1 degradation. (**F**) **Deneddylase activity on the Cullin1-Nedd8/Rbx1.** Substrate hydrolysis (1 µM) is shown as a function of time for 4 nM enzyme (CSN, triangle; CSN5^ΔC,WT^/CSN6^ΔC^, square). For context, CSN5^ΔC,R106T^ alone at 900 nM concentration produces less than 30% of deneddylated Cullin1 for 120 min reaction (data point not shown for clarity). Data information: N.D., not significant; -, parameters not measured; error bars  =  s.d.; experiments done at least twice.

The expression and purification of human CSN5 1–257 fragment, in the WT and mutant forms were performed as previously described in [19]. The human CSN6 cDNA was subcloned in pGEX-6P1 vector (Novagen). The CSN6 31–211 fragment was found to be solubly expressed in bacteria and was selected for further work. The 1–257 and 31–211 fragments of CSN5 and CSN6, corresponding to their MPN domains together with N- and C-terminal appendices for CSN5 and a C-terminal extension for CSN6, as illustrated in Figure 1A, are referred to as CSN5^ΔC^ and CSN6^ΔC^, respectively, in the rest of the manuscript. Expression of CSN6^ΔC^ and its variants was performed in *E. coli* BL21pLysS cells (Novagen) in LB or ^15^NH_4_Cl-supplemented M9 minimal expression medium, as required for NMR experiments. Protein expression was carried out overnight at 18°C and the following purification buffer (20 mM Tris pH 7.5, 150 mM NaCl, 0.01% monothioglycerol) was used throughout the purification. A standard purification protocol for GST-tagged proteins was used, including a first glutathione sepharose affinity step, a size exclusion chromatography step and a second affinity step to remove contaminating GST from the purified protein. Purified CSN6^ΔC^ was concentrated to 8 mg mL^−1^ and stored at −80°C until further use. Production of pro-Nedd8 from the pOPIN-E-pro-nedd8 vector (from M. Banfield), was carried out following the protocol described in [24]. Site directed mutagenesis performed in this work were done with the QuikChange Lightning Site-Directed mutagenesis kit (Stratagene) and verified by DNA sequencing. Production and purification procedures for the CSN and Cullin1-Nedd8/Rbx1 were carried out following the protocols described in [8].

### Isothermal titration calorimetry

Isothermal titration calorimetry (ITC) experiments were carried out on a MicroCal ITC_200_ microcalorimeter (GE Healthcare, Piscataway, NJ) at 20°C. The protein samples were all buffer exchanged using PD-10 desalting column (GE Healthcare) into the ITC buffer (20 mM Na MES pH 6.5, 75 mM NaCl). Protein concentrations were measured using a NanoDrop 1000 spectrophotometer (Thermo Scientific, Wilmington, DE), with the following extinction coefficients: 56,840 M^−1^cm^−1^ and 18,450 M^−1^cm^−1^ for CSN5^ΔC^ and CSN6^ΔC^, respectively. Titration of CSN5^ΔC^ (WT and variants; 100 µM) in the cell (200 µL) was performed by sequential addition of CSN6^ΔC^ (WT and variants; 1 mM; 30 injections of 1.8 µL). Integrated raw ITC data were fitted to a one site nonlinear least squares fit model using the MicroCal Origin plugin as implemented in Origin 9.1 (Origin Lab) after the control experiments (titration of the ligand from the syringe into the buffer) were subtracted.

### Fluorescence anisotropy

To obtain quantitative information about the affinity of CSN5^ΔC^ to Nedd8, fluorescence anisotropy measurements were performed at 28°C, using a TECAN safire^2^ plate reader (Tecan). The experiments were performed in 20 mM Hepes pH 7.5 and 75 mM NaCl. Alexa Fluor 488 fluorophore (Life Technologies) was used to label Nedd8 through a standard amine coupling procedure. The fluorescence was measured using excitation and emission wavelengths of 495 nm and 519 nm, respectively. CSN5^ΔC,WT or.R106T^ at varying concentrations (0–600 µM) was incubated in the presence of 4 nM Alexa Fluor 488-Nedd8. The same procedure was followed in the presence of CSN6^ΔC^ where a 1∶1 ratio of CSN5^ΔC^/CSN6^ΔC^ was used to make the complex at the indicated concentrations. For each data point the measurement was done in triplicates and the recorded data corresponds to the average anisotropy. The data obtained were plotted and analysed using the IgorPro software. The Hill Equation was used for fitting the data in order to be able to deduct an estimate of the apparent equilibrium dissociation constant (K_D_) of each complex between the protein or protein complex and Alexa Fluor 488-Nedd8. As there exists no evidence of cooperativity, in all cases, the data were fit with values of cooperativity equal to 1.

### Isopeptidase assays

Activity assays have been carried out on synthetic and physiological substrates. A synthetic substrate, namely Nedd8-AMC (Nedd8 protein conjugated to a 7-amino-4-methyl-coumarin molecule (AMC); from Boston Biochem.), and one physiological substrate (Cullin1-Nedd8/Rbx1) were used to assess the catalytic activities of different proteins and complexes used in this study. For the Nedd8-AMC substrate, initial rate measurements were carried out at 37°C by following the increase of fluorescence intensity (λ_excitation_  = 380 nm; λ_emission_  = 460 nm), in non-binding surface, flat-bottom, black 384-well plates (Corning), in a volume of 50 µL (reaction buffer (50 mM Tris-HCl pH 7.5, 50 mM NaCl)) on a Tecan safire^2^ plate reader. The following experimental conditions were used: 0.5 to 6.0 µM Nedd8-AMC in reaction buffer, 4 nM CSN5^ΔC^/CSN6^ΔC^ binary complex (110 nM CSN5^ΔC^ and 200 nM CSN6^ΔC^ were mixed prior to reaction and the concentration of the binary complex was calculated using the value of the apparent K_D_ value determined by ITC), 200 nM CSN5^ΔC^ alone or 23 nM CSN. In the case of saturating concentrations of Nedd8-AMC, initial rate data were fitted to the Michaelis-Menten equation using least square analysis to determine k_cat_ and K_M_ in the Prism software (GraphPad). When saturating concentrations of Nedd8-AMC were not reached, the value of k_2_, corresponding to an approximate value of the k_cat_/K_M_ ratio was obtained by linear fitting of initial rate data.

### Deneddylation assays

The CSN5^ΔC,WT^/CSN6^ΔC^ complex and recombinant CSN, were diluted to 4 nM in reaction buffer (20 mM Tris-HCl pH 7.5, 50 mM NaCl) and incubated at 37°C in the presence of 1 µM Cullin1-Nedd8/Rbx1. A time course (0 to 120 minutes) was recorded for each reaction in independent duplicates. One time point was performed for CSN5^ΔC,R106T^ (900 nM) for 2 hours incubation time. The reactions were stopped in SDS sample buffer and analysed on Tris-tricine gel stained in Instant Blue solution (Expedeon, UK). Bands of Cullin-Nedd8/Rbx1were detected and quantified using Carestream Molecular Imaging instrument connected to the Gel Logic 2200PRO software (Equilab, Whitestone, NY).

### Pro-Nedd8 processing assays

This assay conditions were as follows: reaction buffer (20 mM Tris-HCl pH 7.5, 50 mM NaCl); 1 µM pro-Nedd8 WT or variants; CSN5^ΔC^ (WT or variants), alone or the CSN5^ΔC^/CSN6^ΔC^ complex was diluted to 900 nM (for CSN5^ΔC^ alone) or to 4 nM (for the dimer: 110 nM CSN5^ΔC^ and 200 nM CSN6^ΔC^). A time course at 37°C was performed in independent duplicates for each reaction. Samples were analysed as described for deneddylation assays.

### Crystallization, data collection and structure determination

The coordinate and structure factor files have been deposited in the PDB under the code 4QFT. Purified CSN6^ΔC^ at 8 mg mL^−1^ was subjected to crystallisation trials using commercial screening kits. Crystals grown at 18°C, in 0.2 M Tri-Sodium citrate, 0.1 M Bis-Tris propane pH 6.5 and 20% PEG3350, were harvested, cryo-protected in the crystallisation solution supplemented with 10% glycerol and flash-frozen in liquid nitrogen. A dataset was collected at a 1.8 Å resolution at ESRF ID29 beamline and processed using MOSFLM and SCALA from CCP4 suite [25]. The structure was solved by molecular replacement using Phaser with the chain A of human Rpn8^ΔC^ (residues 1–187) as the search model (PDB code 2O95) [26]. The use of human Rpn8 fragment structure as the search model for molecular replacement is supported by the fact that (i) the boundaries of the human CSN6^ΔC^, on which we worked, better correspond to those of human Rpn8 than to those of Drosophila CSN6^ΔC^; (ii) the structure of *Drosophila melanogaster* CSN6^ΔC^ that lacks the C-terminal portion of the MPN domain displays an atypical strand orientation at its C-terminus; (iii) the crystal structure of the human Rpn8^ΔC^ template model is at the highest resolution (1.95 Å). The initial model was built by ARP/wARP into the electron density map [27]. Further refinement procedure was carried out by alternate cycles of manual rebuilding and REFMAC refinement. Data collection and refinement statistics are compiled in Table S1.

### NMR

Uniformly ^15^N-labelled CSN6^ΔC^ protein samples were dissolved in 50 mM Tris buffer, 150 mM NaCl (pH adjusted to 6.8) in 90% H_2_O/10% D_2_O. In all experiments, the ^1^H carrier was centred on the water resonance and a WATERGATE sequence [28], [29] was incorporated to suppress solvent resonances. CSN6^ΔC^ assignment. Spectra were acquired at 298K and pH 6.8, with a 600 µM CSN6^ΔC^ protein sample on a 700 MHz Avance Bruker spectrometer equipped with triple-resonance (^1^H, ^15^N, ^13^C) z-gradient cryo-probe (Figure S1A). NMR data were processed using GIFA (v 4.0) [30], and Topspin (v. 2.1). The 3D ^1^H-^15^N-NOESY-heteronuclear single quantum coherence spectroscopy (HSQC) spectrum was analysed using strip-plots with manual reordering of the sequential stretches according to the main chain assignment strategy [31], [32], [33]. Side chain assignments were carried out using the ^1^H-^15^N-TOCSY-HSQC spectrum. The first two residues Gly-Pro in the N-terminal tag were not assigned. For the remaining of the sequence, assignments were obtained for ^15^N, HN and Ha atoms (excluding Pro residues, and A71, L89, D101 and H160 residues). The overall assignment completeness for ^1^HN-^15^N resonances was 98% (Tables S2, S3). CSN5^ΔC^/CSN6^ΔC^ complex. An HSQC spectrum at 308 K was acquired starting from a ^15^N-labelled CSN6^ΔC^ sample at 100 µM (Figure S1B). After addition of 200 µM unlabelled CSN5^ΔC^ to the ^15^N-labelled CSN6^ΔC^ sample, another HSQC was recorded (Figure S1B). The assignments of CSN6^ΔC^ at 308K were derived from those at 298K, by recording an intermediate CSN6^ΔC^ HSQC spectrum at 305K.

### Protein-protein docking and modelling

CSN5^ΔC^ and CSN6^ΔC^ models were treated as rigid bodies and all six rotational and translational parameters were fully explored in the *ab initio* docking program ZDOCK using 6° sampling on Euler angles. A list of 30 ‘passive’ solvent-accessible residues of CSN6^ΔC^ excluded from the interface with CSN5^ΔC^ derived from the analysis of non-shifted CSN6^ΔC^ peaks in HSQC experiments in the presence of CSN5^ΔC^ was incorporated in the filtering option of the ZDOCK procedure (Figure S1B; Table S4). Among the 54,000 models generated, the top 2,000 complexes, ranked according to ZDOCK were further analyzed. The starting structures for human CSN5^ΔC^ and CSN6^ΔC^ proteins were the crystal structures solved at 2.6 Å and 1.8 Å resolution, respectively. Two docking pairs of protein models were used for docking simulations: (i) the CSN5^ΔC^ (chain A, PDB code: 4F7O), with Ins-1 segment (residues 98–132) fitted to the corresponding ubiquitin-liganded AMSH-LP Ins-1 structure (AMSH-LP structure corresponds to the MPN domain: residues 264–436; PDB code 2ZNV) using MODELLER9 program, followed by manual fitting (to avoid steric clash with the C-terminal LRGG residues of ubiquitin from AMSH-LP distal ubiquitin), combined with the 38–191 CSN6 fragment (α4 deletion; referred to as CSN6^ΔC,Δα4^); and (ii) the same CSN5^ΔC^ model with the N- and C-terminal appending segments removed (residues 2–31 and 232–257; referred to as CSN5^ΔC,Δ2–31,Δ232–257^) combined with CSN6^ΔC,Δα4^ (residues 38–207). We subsequently filtered and re-ranked the 2000 hits using a dedicated procedure incorporating additional experimental constraints in order to identify the correct docking conformations. These constraints were derived from mutagenesis and biochemical data (CSN6 H44, V115 residues and CSN5 E115, Y116, Y120 residues were contributing to the CSN5/CSN6 interface). Additional information was also incorporated in this filtering procedure: primary sequence conservation data for both CSN5 and CSN6 calculated using the PAT server (sequence conservation calculated for each residue in the aligned sequences, by pairwise comparisons using a BLOSUM62 matrix-derived substitution probability score and averaged/normalized for each position to have a 100% value for absolute conservation) [34], total buried area in the dimer interface (calculated using the NACCESS program (http://www.bioinf.manchester.ac.uk/naccess), surface shape complementarity (obtained by SC program [25], [35]) and interface parameters derived from the PISA program [36]. Docking simulations were performed on Intel Linux cluster platforms. The low frequency normal modes of CSN5^ΔC^ were computed with WEBnm@ server [37], providing information on regions with slow collective motions. The six first non-trivial modes were analyzed.

### CX-MS

Roughly 100 µl of 1.2 mg/ml CSN sample were cross-linked at 1 mM disuccinimidyl suberate d0/d12 (DSS, Creative Molecules Inc.), followed by tryptic digestion and enrichment for cross-linked peptides, essentially as described [38]. LC-MS/MS analysis was carried out on an Orbitrap Elite mass spectrometer (Thermo Electron, San Jose, CA) and data were searched using *xQuest* [39] in iontag mode with a precursor mass tolerance of 10 ppm. For matching of fragment ions tolerances of 0.2 Da for common-ions and 0.3 Da for cross-link ions were used. False discovery rates (FDR) of cross-linked peptides were assigned using *xProphet (version 2.5.2)* [40]. Cross-linked peptides were identified with a delta Score <0.95 and a linear discriminant (ld) score >25 and additionally analyzed by visual inspection in order to ensure good matches of ion series on both cross-linked peptide chains for the most abundant peaks.

### Revisiting the topology of CSN

The starting point of the modelling work was the assembly model generated by [8]. To the individual subunit models of the initial CSN model, were substituted updated models of the following subunits generated by Phyre [41]: CSN1 (modelled on a partial crystal structure of the *Arabidopsis thaliana* (*At*) protein; PDB code 4LCT; [42]); CSN5 (modelled on the CSN5^ΔC^ crystal structure; PDB code 4F7O; [19]); CSN6 (modelled on the CSN6^ΔC^ crystal structure; this work); CSN7 (modelled on a partial crystal structure of the *At* protein; PDB code 3CHM; [43]); CSN8 (modelled on the crystal structure of eIF3k; PDB code 1RZ4; [44]). First the CSN5^ΔC^/CSN6^ΔC^ dimer model was placed in the EM density map and its position further refined using the CSN5^ΔC^-Nedd8/CSN6^ΔC^ ternary model. The remaining newly modelled CSN subunits were placed in the density at their respective positions, as established by [8], leaving an unoccupied portion in the electron density. Parallel with the 26S proteasome lid suggested that the C-termini portions of the CSN subunits might occupy this portion. We therefore manually placed the modelled C-termini of the CSN subunits in this density, exploiting inter-subunit cross-links (Table S5). In the last step of modelling, a Molecular Dynamics Flexible Fitting (MDFF) approach was used to improve this initial fit and the quality of the final assembly model [45]. To do so, the various CSN subunits were first rigidly fitted into the EM map (EMD 2173) [8], using the collage program in SITUS package [46]. We used the MDFF procedure with implicit solvent and optimized strength of structural restraints and steering forces [47], together with additional Cα-Cα Lys distance restraints to constrain the experimental inter-subunit cross-linked lysine residues. 5000 cycles of minimization were followed by 100,000 cycles of restrained steered MD. The accuracy of the model was assessed by local correlation between the model and the EM map, and by analysis of the Cα-Cα distance between cross-linked Lys residues.

## Results

### The MPN domains of CSN5 and CSN6 form a stable heterodimer

Previous structural studies of the CSN suggested that CSN5 and CSN6 interact [8], [48]. We therefore set out to quantify the association between the CSN MPN subunits. The association of the 1–257 and 31–211 domains of CSN5 and CSN6, amenable to soluble bacterial expression, referred to as CSN5^ΔC^ and CSN6^Δ^, respectively (Figures 1A, S2) was probed by ITC experiments. The data revealed a K_D_ of 6±1 µM for the MPN heterodimer (Figure 1B,C). Compatible with these findings, significant changes in the HSQC spectra comprising shifts, intensity changes and disappearance of peaks were observed when CSN5^ΔC^ was added to ^15^N-uniformly labelled CSN6^ΔC^ (Figure S1B). Moreover, binding of CSN6^ΔC^ to a conformationally-relaxed CSN5^ΔC^ variant, in which the residue arginine 106 of the Ins-1 is substituted by a threonine (CSN5^ΔC,R106T^) [19] was probed by ITC. In this previous structural, biochemical and computational analysis of CSN5^ΔC^, the Ins-1 region was identified as an important region for the activation state of CSN5^ΔC^. More specifically, in the WT inactive form of CSN5^ΔC^, the Ins-1 appears conformationally constrained and folded back onto the zinc-binding site. The conformational relaxation of the Ins-1, allowing an activity gain of the enzyme, was achieved *in silico* by loss of a salt bridge between the arginine 106 (R106) and the aspartate 151 (D151) upon substitution of R106 by a threonine, as shown in molecular dynamics simulations. This was mirrored *in vitro* by an activation of the enzyme upon Ins-1 conformational relaxation resulting from the un-anchoring of Ins-1 from the zinc-binding site. ITC measurements indicated a comparable affinity between CSN6^ΔC^ and the conformationally-relaxed CSN5^ΔC,R106T^ variant, to that of CSN5^ΔC,WT^/CSN6^ΔC^ (Figure 1C).

### CSN6^ΔC^ confers increased Nedd8 affinity and isopeptidase activity to CSN5^ΔC^

The CSN catalytic subunit lacks enzymatic activity in a stand-alone form, due to the auto-inhibitory position of Ins-1 [17], [19], [48]. We hypothesized that, while CSN5 by itself has a very low affinity for Nedd8, upon activation by an unknown factor, the Ins-1 region rearranges to form part of a Nedd8 recruitment site groove. Interestingly, MPN- subunits of the 26S proteasome lid and of BRCC36-containing complexes are able to activate their otherwise inactive catalytic subunits, Rpn11/POH1 and BRCC36, respectively [20]. Having shown that the N-terminal MPN domains of CSN5 and CSN6 heterodimerize, we went on to evaluate the effect of CSN6^ΔC^ on CSN5^ΔC^'s affinity for Nedd8 and its isopeptidase activity on substrates ranging from protein to a physiological complex.

First we utilized a fluorescence anisotropy assay to assess the binding affinity of Alexa Fluor 488-labelled Nedd8 for CSN5^ΔC^ variants (Figure S3). Consistent with our hypothesis, CSN5^ΔC,WT^ alone displays the weakest affinity for Nedd8 (K_D_ = 320±59 µM), but conformational relaxation of the Ins-1 region in CSN5^ΔC,R106T^ increases the affinity to an apparent K_D_ of 202±25 µM. Importantly, the CSN5^ΔC,WT^/CSN6^ΔC^ (or CSN5^ΔC,R106T^/CSN6^ΔC^) complex has a similar affinity for Nedd8, with an apparent K_D_ of 179±17 µM (or 144±22 µM). Notably, CSN6^ΔC^ alone does not appear to greatly contribute to Nedd8 binding (Figure S3A).

Next the catalytic properties of the enzymes were investigated on Nedd8-AMC (Figure 1D). Consistent with previous data [19], 200 nM CSN5^ΔC,R106T^ hydrolysed Nedd8-AMC at a detectable rate, whilst CSN5^ΔC,WT^ showed no activity. As saturating concentrations of the substrate were not reached, we used the available titration data of CSN5^ΔC,R106T^ to determine a k_app_ value of 57±6 M^−1^s^−1^ from the linear fitting of the initial rate data. The CSN5^ΔC,WT^/CSN6^ΔC^ binary complex at a 4 nM concentration, obtained by mixing 110 nM CSN5^ΔC,WT^ and 200 nM CSN6^ΔC^, displayed robust catalytic efficiency (k_app_ value of 59×10^3^±1×10^3^ M^−1^s^−1^), although the activity of CSN5^ΔC,WT^/CSN6^ΔC^ complex was not saturable at 6 µM substrate concentration and therefore determination of the k_cat_/K_M_ ratio was not possible (Figure 1D). For comparison with the data on the binary complex, we measured the ability of the holo-CSN to hydrolyse Nedd8-AMC, which showed saturation by the substrate (Figure 1D). CSN possesses an estimated k_cat_/K_M_ ratio value of 17×10^3^±1×10^3^ M^−1^s^−1^ towards Nedd8-AMC.

We subsequently studied the hydrolytic activity of CSN and of the CSN5^ΔC^/CSN6^ΔC^ dimer on the physiological substrate Cullin1-Nedd8/Rbx1. The holo-CSN was very efficient at hydrolysing the isopeptide bond between Nedd8 and Cullin1, as reported in [49] (Figure 1E,F). Within less than 10 minutes, most of the Cullin1-Nedd8 substrate at 1 µM, was hydrolysed by 4 nM CSN, as determined by gel shift assay (Figure 1E). Comparatively, the activity of 4 nM CSN5^ΔC,WT^/CSN6^ΔC^ dimer on 1 µM Cullin1-Nedd8 is lower, with a modest proportion of the substrate processed in the same period (Figure 1E,F). For context, the processing of 1 µM Cullin1-Nedd8 by 900 nM CSN5^ΔC,R106T^ alone (i.e. over 200-fold molar excess of enzyme compared to the CSN5^ΔC^/CSN6^ΔC^ conditions) after 120 min incubation revealed that the R106T variant alone has a reduced capacity to hydrolyse Cullin1-Nedd8 compared to the binary CSN5^ΔC,WT^/CSN6^ΔC^ complex.

These findings may imply that the heterodimer constituted by the CSN5/CSN6 MPN domains in the stand-alone state possibly correspond to the minimal catalytic centre, significantly contributing to the isopeptidase activity of human CSN, but with low efficiency on a CSN physiological substrate. As CSN displays significantly higher deneddylase activity than the CSN5/CSN6 MPN complex on a physiological cullin substrate, this could suggest that the optimal activity of the CSN5/CSN6 MPN domains would only be revealed in the context of the holo-CSN complex. These data suggests a hierarchy in the catalytic activity over the enzymatic system and the substrate type, with the inactive CSN5^ΔC,WT^ form, CSN5^ΔC,R106T^ that has basal activity, the MPN heterodimer alone that has robust isopeptidase activity on non-physiological substrates and the CSN5/CSN6 heterodimer in the context of CSN that recapitulates strong activity on both non-physiological and physiological substrates.

### CSN6^ΔC^ crystal structure resembles that of Rpn8^ΔC^

**Figure 2:**
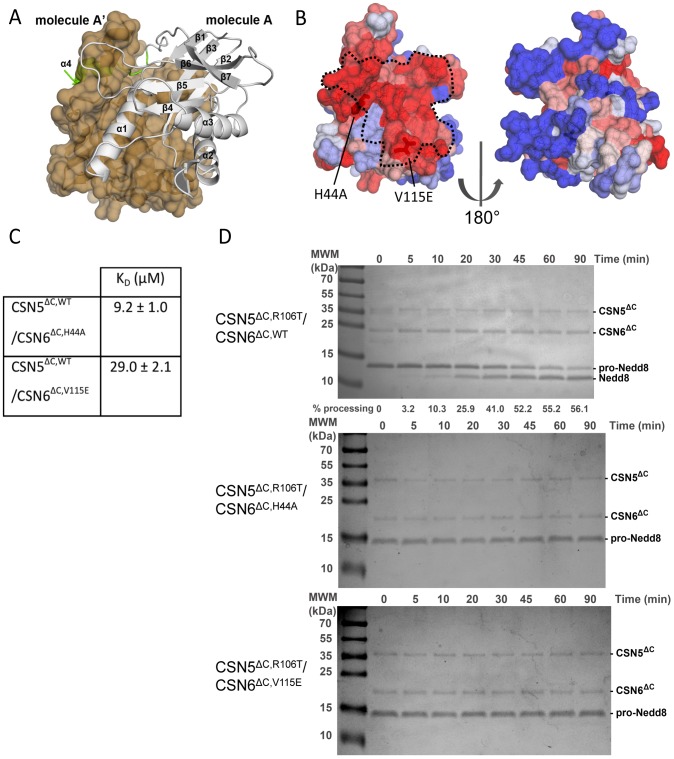
CSN6^ΔC^ binds to CSN5^ΔC^. (**A**) **Human CSN6^ΔC^ displays a classical MPN fold**. The crystallised fragment of human CSN6 contains an MPN core (white ribbon), completed by a C-terminal extension (green ribbon). The asymmetric unit contains one molecule (A); the 2-fold crystallographic symmetry-related one, A′ is shown in beige as a surface. (**B**) **CSN6^ΔC^ binding to CSN5^ΔC^ engages a conserved surface.** CSN6 sequence conservation is displayed on the molecular surface of CSN6^ΔC^ with a colour coding blue to red from variable to conserved, respectively, as calculated with the PAT server [34]. The dotted line corresponds to the surface fingerprint of CSN5^ΔC,Δ2–31,Δ232–257^ in the docking model of CSN5^ΔC^/CSN6^ΔC^. (**C**) **K_D_ values of CSN5^ΔC^**/**CSN6^ΔC^ variant pairs obtained from ITC data.** (**D**)** Pro-Nedd8 processing by CSN5^ΔC^.** Time course assay of pro-Nedd8 (1 µM) processing by CSN5^ΔC,R106T^/CSN6^ΔC^ (top; 4 nM, obtained from mixing 150 nM CSN5^ΔC,R106T^ and 200 nM CSN6^ΔC^), CSN5^ΔC,R106T^/CSN6^ΔC,H44A^ (middle; 4 nM) CSN5^ΔC,R106T^/CSN6^ΔC,V115E^ (bottom; 4 nM), were analysed on Coomassie stained SDS-PAGE gels. The time course assay was analysed on Coomassie stained 15% Tris-tricine SDS-PAGE. Quantification of pro-Nedd8 hydrolysis is specified, when possible, at the bottom of the gel. Quantification of the pro-Nedd8 processing was carried out as detailed in the Material and Methods section. Although CSN5^ΔC,WT^ in the presence of CSN6^ΔC^ displays robust activity on a range of substrates, as illustrated in Figure 1D,E,F, its level remains lower to that of CSN5^ΔC,R106T^ in the presence of CSN6^ΔC^. For the pro-Nedd8 gel shift assay, as illustrated in this figure, for detection purpose, it was advantageous to use the best enzymatic system available.

To gain additional insights into the molecular mechanism of CSN5 activation by CSN6, the crystal structure of the MPN^−^ core fragment was determined by molecular replacement at 1.76 Å resolution, using the human Rpn8^ΔC^ orthologue as the search model (PDB code 2O95; Table S1). The CSN6^ΔC^ structure encompasses the typical MPN domain fold composed of a central β-sheet made of seven strands decorated by three α-helices and a small anti-parallel β-sheet in the Ins-1 region. The MPN core is extended by a C-terminal appendix (residues 192 to 208) that includes a small helical portion (α4 201 to 207; Figure 2A). The asymmetric unit contains one CSN6^ΔC^ molecule, but the crystallographic 2-fold symmetry operator generates an α-helix swapping-stabilised dimer, involving the C-terminal α4 helices (Figure 2A). The MPN domain dimerisation is structurally well documented, but, until recently, all the MPN dimers identified were mediated via different interfaces [14]. Interestingly, despite their sequence conservation (Figure S4A), the homodimeric organisation observed in human CSN6^ΔC^ is different from that in a CSN6 51–187 fragment from *Drosophila melanogaster* [16], but identical to the human Rpn8^ΔC^ and to the budding yeast Rpn11^ΔC^/Rpn8^ΔC^ heterodimer crystal structures, comprising the Rpn8 2–178 or 1–176 and Rpn11 2–239 or 1–220 fragments [21], [22], [50] (Figure S4 B–G). Human CSN6^ΔC^ dimer showed high structural homology with swapped Rpn8^ΔC^ dimer, as illustrated in Figure S4B,C,F,G. 304 equivalent Cα atoms were superimposed with a rmsd of 1.69 Å and a sequence identity of 23% using LSQMAN program [51]. The same comparison of the monomeric form of human CSN6^ΔC^ with human Rpn8^ΔC^ and Drosophila CSN6^ΔC^ gave rmsd values of 1.64 Å for 153 Cα pairs and 1.35 Å for 84 Cα pairs, respectively.

### A highly conserved surface of CSN6^ΔC^ could mediate the association with CSN5^ΔC^

Crystallisation of the human CSN5^ΔC^/CSN6^ΔC^ heterodimer has so far been unsuccessful, in spite of extensive trials. Therefore, to characterise further this complex, we probed the association of CSN5^ΔC^ and CSN6^ΔC^ by NMR to help define the regions responsible for the interaction. Because of the difficulties to unambiguously ascribe CSN6^ΔC^ residues affected by CSN5^ΔC^ (owing to size and poor solubility of the binary complex), instead we extracted, through ^15^N-CSN6^ΔC^ HSQC experiments, the residues that were *not* perturbed upon addition of CSN5^ΔC^ (20% of the residues; Table S4). Although not unambiguous, these NMR restraints were useful in combination with sequence conservation pattern and crystal packing information to delineate a CSN6^ΔC^ surface potentially impacted by the addition of CSN5^ΔC^. The CSN6^ΔC^ surface engaged in the binding to CSN5^ΔC^ most likely corresponds to the region comprised between the α1/α2 helices, as delineated in Figure 2B with dotted grey line. Indeed the functional significance of this particular surface is strengthened by the facts that: (i) it is a highly conserved surface among CSN6 family members (Figures 2B,S4A) and (ii) it corresponds to the homodimer interface found in human CSN6^ΔC^ and Rpn8^ΔC^ crystal structures, as well as to the heterodimer interface found in budding yeast Rpn11^ΔC^/Rpn8^ΔC^ (Figures 2A,S4B,C,F,G) [21], [22].

To further validate this interaction surface, we mutated two CSN6^ΔC^ residues, H44 to an alanine and V115 to a glutamic acid, chosen for their high conservation and their positions on the putative interaction surface, and determined the effect of these mutations on the association with CSN5^ΔC^ by ITC and activity measurements (Figures S4A,2B,C,D). The H44A variant showed a slight reduction in heterodimerisation compared with the WT control, whereas the V115E variant showed a significant 5-fold reduction in binding (Figures 2C,1C). It is noteworthy that, although the interaction between CSN5^ΔC^ and CSN6^ΔC^ was impaired by the H44A or to a stronger extent by the V115A substitution, the effect of these variants remains modest (2 to 5-fold). The interface mediating the CSN5^ΔC^/CSN6^ΔC^ complex obtained from the molecular docking encompasses 1,430 to 1,695 Å^2^, depending on the type of heterodimer chosen. Prediction of ‘hot spot’ residues making a dominant contribution to the binding free energy is a difficult task. Here the buried surface is sizeable and could resist a single substitution not targeting a ‘hot spot’, whilst affecting a topological modification that would decrease the activation of CSN5^ΔC^ by the mutated CSN6^ΔC^ form(s). We subsequently probed the hydrolytic activity of the CSN5^ΔC,R106T^/CSN6^ΔC H44A or V115E^ complex on the Nedd8 precursor, pro-Nedd8. Indeed, our *in vitro* work suggested that the CSN5^ΔC^/CSN6^ΔC^ complex is able to process pro-Nedd8. Although CSN5 is an unlikely factor for pro-Nedd8 processing [52], we have exploited it as a reaction tool to substitute for Nedd8-AMC synthetic substrate, in a gel shift assay, where pro-Nedd8 processing was followed by gel quantification of the appearing Nedd8 band (Figure 2D). Using this pro-Nedd8 gel shift assay, we showed that the hydrolytic activity of the CSN5^ΔC,R106T^/CSN6^ΔC,H44A or V115E^ variant complexes was entirely compromised (Figure 2D).

Taken together and in the context of sequence conservation and of NMR HSQC data, these results suggest an implication of the residues H44 and V115 in CSN5^ΔC^/CSN6^ΔC^ binding - although these residues are possibly not located in a interfacial ‘hotspot’ - and in CSN6^ΔC^-mediated activation of CSN5^ΔC^, reinforcing the view that the CSN5/CSN6 MPN heterodimer is likely to involve a highly conserved surface exploited in other MPN dimers [21], [22], [50]. Further structural confirmation of the involvement of this surface would be required (see Notes from Authors).

### Proposed model of the CSN5/CSN6 MPN domain heterodimer

Having identified a plausible CSN5^ΔC^/CSN6^ΔC^ interaction interface, we attempted to further explore the assembly of the CSN5^ΔC^/CSN6^ΔC^ dimer, molecular docking of the CSN5^ΔC^/CSN6^ΔC^ complex guided by experimental constraints. It is now well established that the incorporation of a few biochemical and NMR-derived constraints in docking methods suffices to determine the structure of a complex to high precision [53].

**Figure 3:**
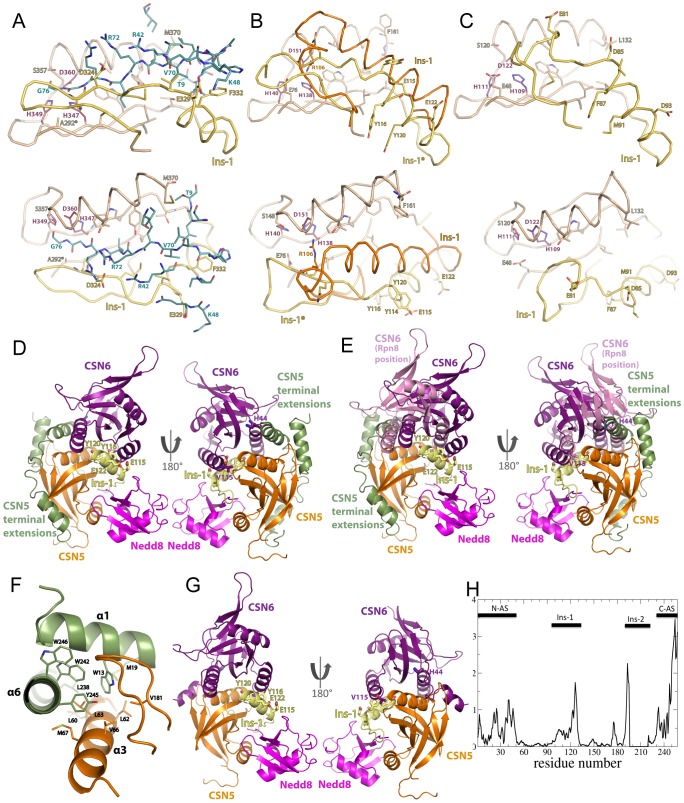
JAMM/MPN^+^-MPN^−^ assembly. (**A,B,C**) **Ins-1 conformation and UBL binding.** Residues discussed in the text are shown in stick mode and the zinc-coordinating residues in purple. Bottom panel: 90° rotated view with respect to the top panel. (**A**) **AMSH-LP MPN Ins-1 bound to distal ubiquitin.** The binding site of AMSH-LP MPN domain to distal ubiquitin (dark blue), as revealed in [18], is represented in beige and in yellow, for the Ins-1 region. The position 292, noted A292*, in AMSH-LP MPN has been substituted to an alanine. (**B**) CSN5^ΔC^ Ins-1 region in the stand-alone form (orange) and modelled on the basis of AMSH-LP MPN Ins-1 conformation (noted Ins-1*, yellow). The zone corresponding to the distal ubiquitin binding site in AMSH-LP MPN (in A) is in beige. (**C**) **Rpn11^ΔC^ Ins-1 region in the context of the Rpn11^ΔC^/Rpn8^ΔC^ complex.** The Rpn11^ΔC^ Ins-1 region, together with the zone corresponding to the distal ubiquitin binding site in AMSH-LP MPN (in A) are presented in yellow and beige, respectively. (**D,E,F,G**) The structures, in ribbon mode, are, unless specified otherwise, coloured as follows: CSN5^ΔC^ or CSN5^ΔC,Δ2–31,Δ232–257^ in orange (N- and C-terminal extensions in green; Ins-1 region in yellow), CSN6^ΔC,Δα4^ in violet, Nedd8 in magenta. When visible, the CSN6^ΔC^ H44 and V115 residues and CSN5^ΔC^ E115, Y116 and Y120 are shown in stick mode. (**D**) **A CSN5/CSN6 model obtained by data-driven docking.** The best CSN5^ΔC^-Nedd8/CSN6^ΔC,Δα4^ pose obtained from the docking procedure is represented. Right panel: 180° rotated view of left Panel. (**E**) **Topology of CSN5^ΔC^**/**CSN6^ΔC^ docking model and Rpn11^ΔC^/Rpn8^ΔC^-like CSN5^ΔC^**/**CSN6^ΔC^.** The best CSN5^ΔC^-Nedd8/CSN6^ΔC,Δα4^ pose obtained from filtering ZDOCK solutions is represented, as in (D). To this model, the CSN5^ΔC^-Nedd8/CSN6^ΔC,Δα4^ model built following the Rpn11^ΔC^/Rpn8^ΔC^ structure has been superimposed. The CSN6^ΔC,Δα4^ structure that is superimposed onto Rpn8^ΔC^ is shown in pink to distinguish it from the CSN6^ΔC,Δα4^ obtained in our docking solution. Right panel: 180° rotated view of left Panel. (**F**) **The N- and C-terminal helices specific to** CSN5^ΔC^. The N- and C-terminal helical extensions of CSN5^ΔC^ are stacked onto the α3 helix and the loop linking the β6–β7 anti-parallel sheet. The residues involved in packing are shown in stick mode. (**G**) **CSN5^ΔC,Δ2–31,Δ232–257^/CSN6^ΔC,Δα4^ docking pose.** The best docking pose using a CSN5^ΔC^ model in which the N- and C-terminal extensions were deleted (CSN5^ΔC,Δ2–31,Δ232–257^) is shown. Right panel: 180° rotated view of left Panel. (**H**) **The normalized squared displacement of the CSN5^ΔC^ Cα atoms in the lowest frequency mode 7 from normal mode analysis.**

To help the docking procedure, a complex composed of CSN5^ΔC^, harbouring an AMSH-LP-like Ins-1 conformation and of Nedd8, was assembled. To do so, we probed the binding of Nedd8 on CSN5^ΔC^ and reciprocally, to investigate whether Nedd8 binds to CSN5^ΔC^ in a mode reminiscent to that of the distal ubiquitin to the MPN domain of AMSH-LP (or AMSH). The choice of the interface residues to be analysed was guided by the structurally characterised AMSH-LP MPN domain/distal ubiquitin association (Figure 3A,B) [18]. Among the two CSN5^ΔC^ Ins-1 glutamate positions probed (E115, E122), the E122D variant kept strong processing activity on pro-Nedd8, whilst the E115D one displayed weak activity (Fig S5A). The importance of F332 in AMSH-LP for the distal ubiquitin binding prompted us to evaluate the role of two aromatic residues in CSN5^ΔC^ Ins-1 region (Y116, Y120) on the hydrolysis of the Nedd8 precursor (Fig S5A). Additionally, effects of four pro-Nedd8 variants (T9M, R42D, K48A, V70D) on processing activity were assessed (Fig S5B). Activity measurement data on the chosen CSN5^ΔC^ and pro-Nedd8 variants showed that the CSN5^ΔC, R106T, E115A, Y116D.or.Y120D^ variants have compromised pro-Nedd8 processing activity and that the pro-Nedd8 ones, namely pro-Nedd8^T9M, R42D, K48A or V70D^, are important for hydrolysis by CSN5^ΔC^, validating that Nedd8 on CSN5^ΔC^ probably orient similarly to the distal ubiquitin and AMSH-LP MPN (and Sst2, the AMSH homolog in yeast) (Figures 3A,B, S5A,B) [18], [54].

To assemble the CSN5^ΔC^-Nedd8 complex we then modelled the CSN5 Ins-1 segment to resemble that of AMSH-LP MPN. Interestingly, as a result of the modelling procedure, CSN5^ΔC^ modelled Ins-1 resembles the conformation of Rpn11^ΔC^ Ins-1 in the binary complex with Rpn8^ΔC^ (Figure 3C). Exploiting the above detailed finding that CSN5^ΔC^ and Nedd8 interaction proceeds through similar surfaces to that of AMSH-LP-distal ubiquitin's (Figures 3A,B, S5A,B), a complex composed of CSN5^ΔC^ displaying an AMSH-LP-like Ins-1 conformation and of Nedd8 was assembled and used to probe the association of CSN5^ΔC^ and CSN6^ΔC^.

Subsequently, molecular docking simulations of the CSN5^ΔC^/CSN6^ΔC^ dimer were performed by the software ZDOCK followed by a tailored post-docking filtering strategy. The simulations resulted in a cluster of similar docking poses with favourable interfacial metrics and satisfying all our experimental data (Figures 3D, S6). In the resulting model, CSN5^ΔC^ and CSN6^ΔC^ are placed in a topology that happens to be close to that observed in the crystallographic human Rpn8^ΔC^ and human CSN6^ΔC^ homodimers (Figures 3D, S4B,C,F,G) [50].

The surfaces engaged in the CSN5^ΔC^/CSN6^ΔC,Δα4^ model are highly similar to those of the Rpn11^ΔC^/Rpn8^ΔC^ complex (Figures 3E, S6). However, superimposition of their MPN^+^/JAMM subunits revealed that the CSN6^ΔC,Δα4^ position on CSN5^ΔC^ surface in our model is rotated and translated, relative to the Rpn8^ΔC^ position on Rpn11^ΔC^ (Figure 3E). This difference is the result of the presence of the CSN5-specific N- and C-terminal helical appendices on each side of the MPN domain (residues 6 to 17 and 235 to 251, respectively). The CSN5 specific N-terminal extension wraps around its MPN core and C-terminal helix (Figure 3F) [19]. For CSN5^ΔC^ and CSN6^ΔC^ to arrange in a Rpn11^ΔC^/Rpn8^ΔC^-like dimer, it would take the CSN5-specific N- and C-terminal α-helices to unstaple from its MPN core. To confirm this, docking carried out on a CSN5^ΔC^ variant lacking the N- and C-terminal helices (fragment 32–232) yielded top scoring solutions corresponding to a pose of the Rpn11^ΔC^/Rpn8^ΔC^ topology (Figures 3G, S6). These helices were shown to be conformationally malleable by normal mode analysis [55] of CSN5^ΔC^, where a subset of low frequency modes shows their propensity to undergo large-scale shear and hinge motions (Figures 3H, S7). These secondary structure elements, stabilised by numerous weak interactions, could be unhooked from the core domain (Figure 3F). This analysis could strengthen the view that CSN5^ΔC^ and CSN6^ΔC^ interact through an architecture reminiscent of the Rpn11^ΔC^/Rpn8^ΔC^ complex.

### The molecular architecture of the intact CSN

**Figure 4:**
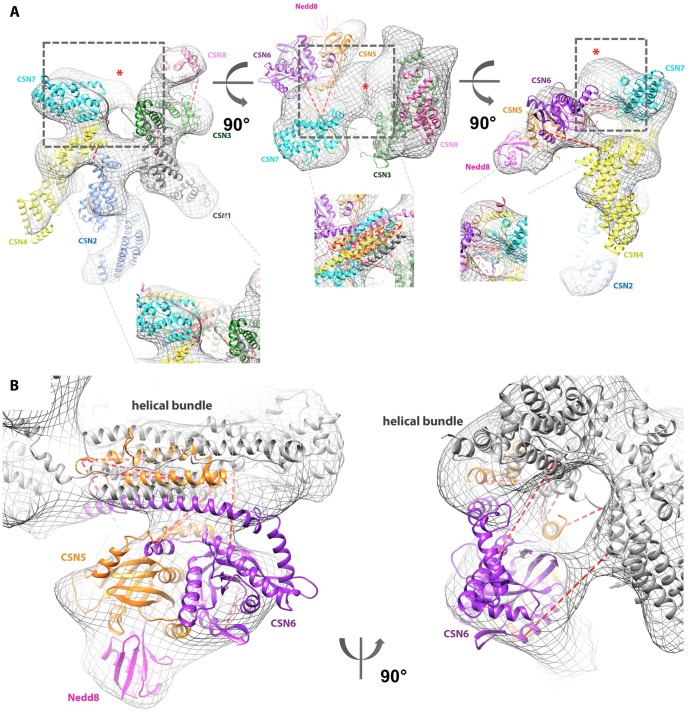
The CSN topology. Inter-subunit cross-links between CSN5 or CSN6 Lys and other CSN subunits (Cα distances <40 Å) are marked with red dotted lines. (**A**) **The CSN assembly revisited**, displayed in the EM density obtained by [8] (grey mesh). A red star indicates an unoccupied electron density region. Close-up views of this region are shown after fitting of the helical bundle. (**B**) **CSN5-Nedd8/CSN6 complex positioned in the EM envelope.** CSN5 is shown in orange, CSN6 in purple and Nedd8 in magenta. CSN subunits (other than CSN5 and CSN6) are shown in white for context.

Finally, we wanted to interpret our CSN5^ΔC^/CSN6^ΔC^ model in the context of the full CSN complex. We adopted a hybrid structural approach, which involved CX-MS of the intact complex [38], cross-link-guided docking of individual subunits and domains and molecular dynamics flexible fitting of the obtained model using MDFF procedure in the CSN density of the previously published CSN-SCF EM map (EMD 2173; Table S5; Figure S8) [8], [47]. For the docking we used the CSN5^ΔC^/CSN6^ΔC^ model generated in this study and for the PCI domains and missing portions of the remaining subunits, we predicted structures using the server Phyre [41] (Figures 4A, S8; Table S6). CSN1 and CSN7 models were largely based on their crystal structures (PDB codes 4LCT, 3CHM, respectively) [42], [43]. The CSN5^ΔC^/CSN6^ΔC^ model described above was fitted as a rigid body (Figure 4A) and docked in the previously assigned EM density portion [8]. However, using our ternary complex model composed of CSN5^ΔC^-Nedd8/CSN6^ΔC^ (Figure 4A) for refinement, resulted in a slightly different orientation. In our model, CSN6^ΔC^ moved to a more peripheral location, relative to the one previously reported [8]. This resulted in a conspicuous density segment left empty in the thus re-docked CSN model (Figure 4A, red star).

Multiple cross-links between the extreme C-terminal sequence portions of peripheral and central subunits, like CSN7-CSN1 or CSN5-CSN1 indicated that these elements contribute a major, previously unrecognised binding interface (Table S5, Figure S8). Importantly, most of these C-terminal elements are predicted to adopt helical structures (Figure S8). This led us to hypothesize that they all join in a central helical bundle in a topology reminiscent of the 26S proteasome lid [23]. To test this hypothesis we generated models for the C-termini of all CSN subunits and arranged them in a compact helical bundle like in [23] (Figures 4A,B, S8). The resulting cluster matched well the corresponding density in shape and size (Figure 4B), although the precise orientation and topology of the helices could not be optimized due to the low resolution of the EM density map. Potential positions of the C-termini portions contributing to the central bundle and of the CSN subunits were refined to best satisfy the set of inter-subunit cross-links providing powerful constraints (21 inter-subunits cross-links between the 8 C-terminus subunits; Table S5).

The local cross-correlation coefficient between the revisited CSN assembly and the EM map (ccc = 0.84) has increased compared to the initial model (ccc = 0.80). Both models for the eight subunits contained the same number of residues (∼2,750), but positioned differently inside the 25 Å resolution map, in the particular case of CSN5/CSN6 and of the C-terminal segments. More significantly, the average distance between lysine Cαs of the 70 identified intra- and inter- cross-links is 32.9 Å (29.5 Å if 4 clear outlier inter-subunit cross-links with distance >60 Å are excluded from the analysis). This results in an improvement over the initial model for which the average distance of the 55 cross-links was ∼38 Å, suggesting that our CX-MS guided procedure has improved the overall subunit topology within the CSN. Illustrative examples of the agreement between identified cross-links with the revisited model, specifically relevant to the helical bundle and to the CSN5/CSN6 dimer with respect to the rest of the edifice are between the C-terminus of CSN1 and CSN3 (distance K467-K418: 23 Å); CSN6 and CSN7 (distance K199-K199: 27 Å); CSN2 and CSN5 (distance K426-K180: 22 Å); CSN5 and CSN7 (distance K180-K199: 32 Å). However it is noteworthy that some of the observed cross-links linkages are difficult to reconcile with the overall topology of the molecular model, as for example the link between CSN1 K97 and CSN2 K415 and between CSN1 K422 and CSN3 K115. Some of these cross-links, for which the distance in the model exceeds the maximal distance that one can expect between two cross-linked residues, may arise from conformational variability within parts of the CSN, as recently demonstrated for subunits Rpn5 and Rpn6 of the 26S proteasome lid, corresponding to CSN4 and CSN2, respectively [12], [56]. It is therefore interesting to speculate, that the observed cross-link distribution may therefore at least partially be influenced by an intrinsically dynamic behaviour of the CSN [8], [11].

## Discussion

By dissecting biochemical and structural elements of the CSN, we have demonstrated that CSN6^ΔC^ binds to the MPN domain of the CSN5 catalytic subunit and this association increases CSN5^ΔC^ affinity for Nedd8 and enhances its hydrolytic activity on a variety of substrates, suggesting that the MPN heterodimer could contribute significantly to the catalytic activity of the human CSN. The activation of an MPN^+^/JAMM protein mediated by a MPN^−^ subunit was described in two other MPN^+^/JAMM-MPN^−^ systems, namely the 26S proteasome lid and BRCC36-containing complexes [20], [21], [22]. This mechanism of activity control of MPN^+^/JAMM enzymes would therefore appear to be a general assembly and regulatory principle of the MPN^+^/JAMM-MPN^−^ containing complexes and as such would constitute an important regulatory mechanism of these complexes [14], [57].

The stimulating role of CSN6^ΔC^ in CSN5^ΔC^ activation demonstrated here should be considered in the light of the data on the CSN reported in [58]. The authors probed the function of CSN6 and its yeast ortholog (Csi1) MPN domain in the human and yeast CSNs, respectively. These experiments, carried out using budding yeast and mammalian CSNs and neddylated cullins purified from yeast and mammalian cells, concluded that only the C-terminal part of CSN6 is necessary for the deneddylase activity. Further investigations are needed to reconcile those results with our observations.

Taken together, our fluorescence anisotropy binding experiments and the activity measurements suggest that CSN6^ΔC^ does not contribute significantly to Nedd8 direct recruitment and activates CSN5^ΔC^ through at least two mechanisms. First, the catalytic activity efficiency can be achieved by an increase of affinity for Nedd8 observed either in the CSN5/CSN6 complex or by the conformational relaxation of the Ins-1 segment. Secondly, the increased activity can also result from a yet unknown mechanism. Indeed, CSN5^ΔC,WT^/CSN6^ΔC^ has a higher catalytic efficiency than CSN5^ΔC,R106T^ alone, although their affinity for Nedd8 is of the same order of magnitude. The contribution of CSN6^ΔC^ to CSN5^ΔC^ activation might therefore not be confined to the enhancement of substrate binding.

Assessment of the CSN5^ΔC^/CSN6^ΔC^ complex and of CSN on different Nedd8-derived substrates provides a molecular ranking of the catalytic activity. The higher hydrolysis rate of CSN on neddylated Cullin1 than on Nedd8-AMC supports the observation that CSN is a cullin deneddylase [7], underlying the contribution of the CSN edifice in the efficient processing of neddylated CRLs. This is therefore in agreement with the observations in [8], that PCI subunits contribute to the recruitment of the CRLs. Indeed EM studies on CSN/CRL supercomplexes highlighted the direct interaction between CSN2, and probably CSN4, and the cullin subunit of the CRL. Taken together, these data show that the CSN is an intricate assembly that is tailored to function on CRLs.

To further probe CSN assembly, we performed molecular docking of the CSN5^ΔC^/CSN6^ΔC^ complex guided by experimental constraints (Figure S6). The MPN domain heterodimerisation appears to be an important aspect in the regulation of several multiprotein complexes in eukaryotes. Vexingly, different MPN-containing domains appear to utilise different dimerisation surfaces [14], although recent structures (budding yeast Rpn11^ΔC^/Rpn8^ΔC^ [21], [22]; human CSN6^ΔC^) exhibit a dimerisation mode consistent with the one originally described for human Rpn8^ΔC^ [50]. Interestingly our model highly resembles the crystal structure of the Rpn11^ΔC^/Rpn8^ΔC^ dimer [21], [22] and the association of CSN6^ΔC^ and of Rpn8^ΔC^ in the direct vicinity of the Ins-1 region of CSN5^ΔC^ and Rpn11^ΔC^, respectively, further underlines the essential role of Ins-1 as a regulatory switch in these enzymes.

Moreover, we explored the molecular assembly of the intact CSN by reinterpreting a published EM density map, using molecular docking of our model of the CSN5^ΔC^/CSN6^ΔC^ complex and experimental data from CX-MS experiments. This work provided an updated model of the CSN structure that comprises the repositioning of the CSN5^ΔC^/CSN6^ΔC^ dimer and a central helical bundle, reminiscent of the 26S proteasome lid and suggests that this bundle may be contributing to the assembly of the CSN [23]. This structural analysis, complementing our work on the MPN domains of CSN5 and CSN6 that highlights the importance of the MPN domain interaction in the catalytic activity, could suggest that the C-terminal regions of CSN5 and CSN6 contribute to anchoring and optimal positioning these subunits within the CSN assembly.

## Supporting Information

Figure S1 **NMR HSQC spectra of ^15^N-CSN6^ΔC^.**
(PDF)

Figure S2 **SDS-Page of CSN5^ΔC^ and CSN6^ΔC^ proteins.**
(PDF)

Figure S3 **Fluorescence anisotropy data on CSN5^ΔC,WT^, CSN5^ΔC,R106T^ and CSN6^ΔC^, on CSN5^ΔC,WT^/CSN6^ΔC^ and CSN5^ΔC,R106T^/CSN6^ΔC^ and their associated apparent dissociation constants.**
(PDF)

Figure S4 **CSN6 sequences and MPN domain structure.**
(PDF)

Figure S5 **Pro-Nedd8 processing by CSN5^ΔC^.**
(PDF)

Figure S6 **Interface residue of the CSN5^ΔC^/CSN6^ΔC^ heterodimer models expressed as the number of atomic contact pairs between residues of the predicted heterodimers.**
(PDF)

Figure S7 **CSN5^ΔC^ normal modes analysis using WEBnm@ server.**
(PDF)

Figure S8 **Linkage map of the CSN subunits obtained from cross-linked CSN followed by MS analysis.**
(PDF)

Table S1 **X ray data collection and refinement statistics for human CSN6^ΔC^ crystal.**
(PDF)

Table S2 **NMR experiments acquired for chemical shift assignments.**
(PDF)

Table S3 **NMR experiments acquired for CSN5^ΔC^/CSN6^ΔC^ mapping.**
(PDF)

Table S4 **Unperturbed CSN6^ΔC^ residues upon addition of CSN5^ΔC^ as evaluated by NMR HSQC.**
(PDF)

Table S5 **Cross-links used in this study.**
(PDF)

Table S6 **Sequences of the CSN subunit models used.**
(PDF)
