# Supplementary material for: Structural and Biochemical Characterization of the Cop9 Signalosome CSN5/CSN6 Heterodimer
Source: PLoS One. 2014 Aug 21;9(8):e105688. doi: 10.1371/journal.pone.0105688 (PMC4140821; doi:10.1371/journal.pone.0105688)
Supplement: Figure S1 — NMR HSQC spectra of 15N-CSN6ΔC. (PDF) [file pone.0105688.s001.pdf]

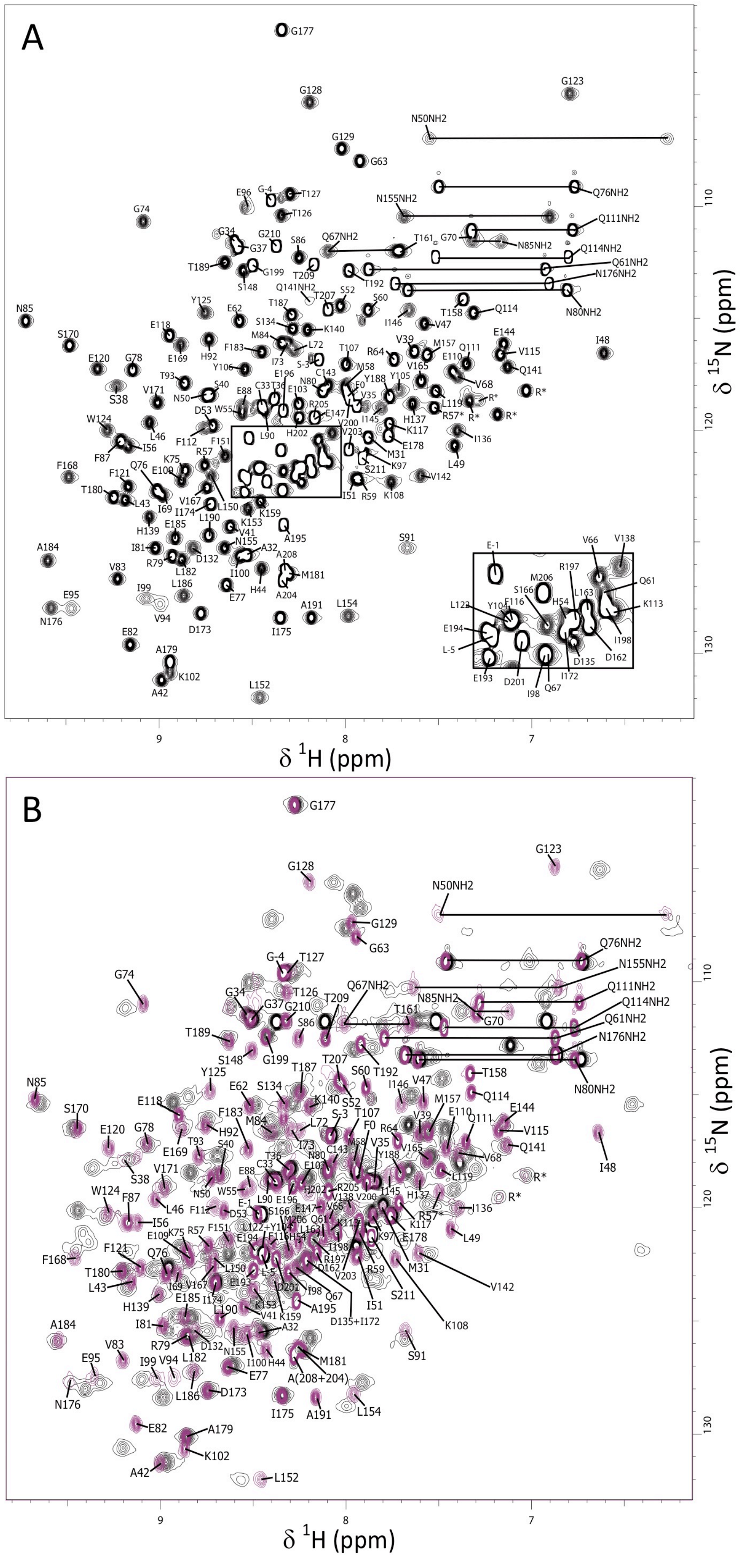

Figure S1. NMR HSQC spectra of ^{15}N-CSN6$^{\Delta C}$. (A) HSQC giving the ^{15}N and ^{1}H chemical shift assignments of CSN6$^{\Delta C}$ at 298K. Negative labels refer to residues from the N-terminal tag, from L-5 to F0. **(B) HSQC spectra of ^{15}N-CSN6$^{\Delta C}$ alone or in the presence of CSN5$^{\Delta C}$ both collected at 308K.** The HSQC spectrum of ^{15}N-CSN6$^{\Delta C}$ is shown in magenta; and in the presence of CSN5$^{\Delta C}$, in grey.
