# Supplementary material for: Structural and Biochemical Characterization of the Cop9 Signalosome CSN5/CSN6 Heterodimer
Source: PLoS One. 2014 Aug 21;9(8):e105688. doi: 10.1371/journal.pone.0105688 (PMC4140821; doi:10.1371/journal.pone.0105688)
Supplement: Figure S3 — Fluorescence anisotropy data on CSN5ΔC,WT, CSN5ΔC,R106T and CSN6ΔC, on CSN5ΔC,WT/CSN6ΔC and CSN5ΔC,R106T/CSN6ΔC and their associated apparent dissociation constants. (PDF) [file pone.0105688.s003.pdf]

A

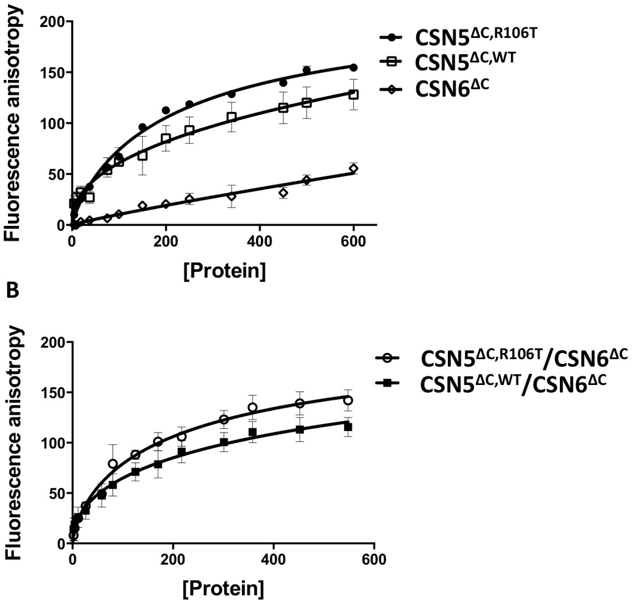

C

Protein or protein complex	Apparent K_D (μM)
CSN5$^{\Delta C,WT}$	320 ± 59
CSN5$^{\Delta C,R106T}$	202 ± 25
CSN5$^{\Delta C,WT}$/CSN6$^{\Delta C,WT}$	179 ± 17
CSN5$^{\Delta C,R106T}$/CSN6$^{\Delta C,WT}$	144 ± 22
CSN6$^{\Delta C,WT}$	N.D.

Figure S3. Fluorescence anisotropy data on (A) CSN5$^{\Delta C,WT}$, CSN5$^{\Delta C,R106T}$ and CSN6$^{\Delta C}$, on (B) CSN5$^{\Delta C,WT}$/CSN6$^{\Delta C}$ and CSN5$^{\Delta C,R106T}$/CSN6$^{\Delta C}$ and (C) their associated apparent dissociation constants. The table displays the apparent K_D (μM) extracted from fluorescence anisotropy titration. The data corresponds to the mean over three independent experiments with the associated standard error. N.D.: as the fluorescence anisotropy values did not reach a plateau, the data did not allow for realistic apparent K_D determination.
