# Supplementary material for: Structural and Biochemical Characterization of the Cop9 Signalosome CSN5/CSN6 Heterodimer
Source: PLoS One. 2014 Aug 21;9(8):e105688. doi: 10.1371/journal.pone.0105688 (PMC4140821; doi:10.1371/journal.pone.0105688)
Supplement: Figure S4 — CSN6 sequences and MPN domain structure. (PDF) [file pone.0105688.s004.pdf]

A

```
DmCSN6    1    --MEQM----EVDVDM-------------SAKPS----TSSSAAAGSSMAVDKTADQNPQ   37
BmCSN6    1    -MSSAD----RIDFEM-------------EV-------EVESASSGPPIEVPNPTSPAPT   35
AgCSN6    1    TMSQPTEEKMDIDNESASATASTSTSASAAMPPTASTSKAASPPAVSDMEVASGSSGKPV   60
BtCSN6    1    ----MA----AT-----------------AA-------AANGTGGSSGMEVDA-------   21
MmCSN6    1    ---MAA----AA-----------------AA-------GANGSGGSSGMEVDA-------   22
HsCSN6    1    -MAAAA----AA-----------------AA-------ATNGTGGSSGMEVDA-------   24
XlCSN6    1    --------------------------------------MAAAASNGNGMEVDV-------   15
DrCSN6    1    ------------------------------------------------------------    0
SmCSN6    1    ------------------------------------------------MEVDI-------    5
OsCSN6    1    ------------------------------------------------MS--APSD--PA    8
AtCSN6    1    ------------------------------------------------------------    0

DmCSN6    38   P----QGNIMAAAGTSGSVTISLHPLVIMNISEHWTRFRAQHG-----------------   76
BmCSN6    36   TPGS-NKSVVVTTATAGSVTVSLHPLVIMNVSEHWTRLKAQEG-----------------   77
AgCSN6    61   PVQPEGRNVMASSATIPSVTCSLHPLVIMNIADHWTRNRAQKS-----------------  103
BtCSN6    22   ---A-VVPSVMASGVTGSVSVALHPLVILNISDHWIRMRSQEG-----------------   60
MmCSN6    23   ---A--VPSVMASGVTGSVSVALHPLVILNISDHWIRMRSQEG-----------------   60
HsCSN6    25   ---A-VVPSVMACGVTGSVSVALHPLVILNISDHWIRMRSQEG-----------------   63
XlCSN6    16   ---A-ALPSVMAQGVTGSVTVALHPLVILNISDHWIRMRSQEG-----------------   54
DrCSN6    1    ----------MASGVTGSVSVALHPLVILNISDHWIRIRSQEG-----------------   33
SmCSN6    6    -----NVDVSNVTSGTGSASVLLHPLVILNISEHWTRNKVKEN-----------------   43
OsCSN6    9    VATHPQAGAAAAASSSSGLTFKLHPLVIVNVSDHHTRVKAQAACS------GDGAS-SAA   61
AtCSN6    1    ----------MAPSSSSGLTFKLHPLVMLNISDHFTRVKTQLNPPAASCATGNGSNNADA   50
                                     *****  *   *  *

DmCSN6    77   ---EPRQVYGALIGKQKGRNIEIMNSFELKTDVIGDETVINKDYYNKKEQQYKQVFSDLD  133
BmCSN6    78   ---FPQTVVGALIGKQKGRNIEVMNSFELVFSMIDGDIIIDRDYYNMKEEQFKQVFSDMD  134
AgCSN6    104  ---SRPLIFGALIGKQKGRNIEVMNSFELKYDIVNDAVVIAMDYYQVKEEQYKQVFSDLD  160
BtCSN6    61   ---RPMQVIGALIGKQEGRNIEVMNSFELLSHTVEEKIIIDKEYYYTKEEQFKQVFKELD  117
MmCSN6    61   ---RPMQVIGALIGKQEGRNIEVMNSFELLSHTVEEKIIIDKEYYYTKEEQFKQVFKELE  117
HsCSN6    64   ---RPVQVIGALIGKQEGRNIEVMNSFELLSHTVEEKIIIDKEYYYTKEEQFKQVFKELE  120
XlCSN6    55   ---RPMQVIGALIGKQEGRNIEVMNSFELLSQINDEKITINKEYYYTKEEQFKQVFKDME  111
DrCSN6    34   ---RAVQVVGALIGKQEGRNIEVMNSFELLFHTVEDQIHIDKEYYYTKEEQFKQVFKEME   90
SmCSN6    44   --SISVTVYGALLGKQEGHHVEISNSFELLLDE--PHMSVNSEFYSTRESQCKQVYPDLD   99
OsCSN6    62   AGGQPPRVFGCVIGVQRGRTVEIFNSFELVLDPVSG--TLDRAFLEKKQELYKKVFPDFY  119
AtCSN6    51   MLLQNPRVYGCVIGLQRGRTVEIFNSFELIFDPALD--TLDRSFLEKKQELYKKVFPDFY  108
                        *   * * *   *  *****                       * *

DmCSN6    134  FIGWYTTGDNPTADDIKIQRQIAAINECPIMLQLNPLSRS--VDHLPLKLFESLIDLVDG  191
BmCSN6    135  FLGWYTTGDAPSERDIAVHRQICDINECPVMLMLNPAGRN--GDQLPVVLYESVIDVVNG  192
AgCSN6    161  FLGWYTTDTVPSEKHINIHKQICEINECPLMLLLDPLNRNMNVRRLPISLYESVIDIVQG  220
BtCSN6    118  FLGWYTTGGPPDPSDIHVHKQVCEIIESPLFLKLNPMTKH---TDLPVSVFESVIDIING  174
MmCSN6    118  FLGWYTTGGPPDPSDIHVHKQVCEIIESPLFLKLNPMTKH---TDLPVSVFESVIDIING  174
HsCSN6    121  FLGWYTTGGPPDPSDIHVHKQVCEIIESPLFLKLNPMTKH---TDLPVSVFESVIDIING  177
XlCSN6    112  FLGWYTTGGTPDPSDIHVHKQVCEIIESPLFLKLNPMTKH---TDLPVSVYESVIDIVNG  168
DrCSN6    91   FLGWYTTGGSPDQSDIHIHKQVCEIIESPLFLKLNPMTKH---TDLPVSVFESVIDIISG  147
SmCSN6    100  IVGWYTTGGAINEKDELFNRQMLELNESLLILKLDPLQTC--GENLPIGVYESVVD---N  154
OsCSN6    120  VLGWYSTGSDVRDTDMQIHKALMDINESPVYLLLNPAINL-SQKDLPVTIYESELHVIDG  178
AtCSN6    109  VLGWYSTGSDATESDMHIHKALMDINESPVYVLLNPAINH-AQKDLPVTIYESEFHVIDG  167
                 *** *                   *      * *         **    **

DmCSN6    192  EATMLFVPLTYTLATEEAERIGVDHVARMTSNESGE-KSVVAEHLVAQDSAIKMLNTRIK  250
BmCSN6    193  RATMLLAPLTYTLAAEEAERIGVDHVARVSSGEAAL-NSLVAEHLTAQRSAIKMLVSRVR  251
AgCSN6    221  QAMMLFVPLVYTLATEEAERIGVDHVARMSNNDTDA-NSTVAEHLLAQYNAIKMLNSRVK  279
BtCSN6    175  EATMLFAELTYTLATEEAERIGVDHVARMTATGSGE-NSTVAEHLIAQHSAIKMLHSRVK  233
MmCSN6    175  EATMLFAELTYTLATEEAERIGVDHVARMTATGSGE-NSTVAEHLIAQHSAIKMLHSRVK  233
HsCSN6    178  EATMLFAELTYTLATEEAERIGVDHVARMTATGSGE-NSTVAEHLIAQHSAIKMLHSRVK  236
XlCSN6    169  EATMLLAELSYTLATEEAERIGVDHVARMTATGSGE-NSTVAEHLIAQHSAIKMLHSRVR  227
DrCSN6    148  EATMLFAELPYTLATEEAERIGVDHVARMTATGTGE-NSTVAEHLIAQHSAIKMLHSRVK  206
SmCSN6    155  DGRVHFRQVLYTLATDEAERIGVDYVARISMSSTDQTSSMTAEHLLGNYQAIQMLSSRLQ  214
OsCSN6    179  SPQLIFVRANYTIETVEAERISVDHVAHLKPSDGGSAATQLAAHLTGIHSAIKMLNSRVR  238
AtCSN6    168  IPQSIFVHTSYTIETVEAERISVDHVAHLKPSDGGSAATQLAAHLTGIHSAIKMLNSRIR  227
                         **    ***** ** **              * **     ** **  *

DmCSN6    251  IVLQYIRDVEAGKLRANQEILREAYALCHRLPVMQVPA--FQEEFYTQCNDVGLISYLGT  308
BmCSN6    252  AVLATVRAIRDGKLQPRPALLREARALANRLPLLTSQQ--FRTHFYNQCNDVALMTYLGT  309
AgCSN6    280  IILAYIKAVENDQLKPNQEILRMAYSLSRRLPIVQNPS--FKEEFYTQSNDVGLITYLGA  337
BtCSN6    234  LILEYVKASEAGEVPFNHEILREAYALCHCLPVLSTDK--FKTDFYDQCNDVGLMAYLGT  291
MmCSN6    234  LILEYVKASEAGEVPFNHEILREAYALCHCLPVLSTDK--FKTDFYDQCNDVGLMAYLGT  291
HsCSN6    237  LILEYVKASEAGEVPFNHEILREAYALCHCLPVLSTDK--FKTDFYDQCNDVGLMAYLGT  294
XlCSN6    228  LILEYVRAAEGGEVPFNHEILREASALCHCLPVLSTDK--FKTDFYDQCNDVGLMSYLGT  285
DrCSN6    207  VILEYVKAVQAGEVPFNHEILREANALCHRLPVLNTLK--FKTDFYDQCNDVGLMAYLGT  264
SmCSN6    215  LIRSYVAAVAAGELPPNRARLREINALVKRIPFMPSSAAEQNDNLYRQANDVCLTALLAS  274
OsCSN6    239  VIHQYLVSMQKGDMPLDNSLLRQVSSLVRRLPAMESEK--FQDDFLMEYNDTLLMTYLAM  296
AtCSN6    228  VLYQHIVAMQKGDKPCENSVLRQVSSLLRSLPAAESEK--FNENFLMEYNDKLLMSYLAM  285
                                   **    *    *                 **  *   *

DmCSN6    309  LTKGCNDMHHFVNKFNMLYDRQGSARRMRGLYY------------------           341
BmCSN6    310  ITKGCNAINQLVNRFNVLYDRQGMGRRMRGLFF------------------           342
AgCSN6    338  LTKVANDMNQLVNKFNVLYDRQAMGRRLRGIFF------------------           370
BtCSN6    292  ITKTCNTMNQFVNKFNVLYDRQGIGRRMRGLFF------------------           324
MmCSN6    292  ITKTCNTMNQFVNKFNVLYDRQGIGRRMRGLFF------------------           324
HsCSN6    295  ITKTCNTMNQFVNKFNVLYDRQGIGRRMRGLFF------------------           327
XlCSN6    286  ITKTCNTMNQFVNKFNILYDRQGIGRRMRGLFF------------------           318
DrCSN6    265  ITKTCNSMNQFINKFNVLYDRQGIGRRMRGLFF------------------           297
SmCSN6    275  ITQGLHTLYGCMVKTSHVIDRRSIPLSVGSGIMDSGSSRFAKVSHSMGNSN           325
OsCSN6    297  FTNCSSTMNELVEKFNATYERSTARRGGRGAFM------------------           329
AtCSN6    286  ITNCTSNMNEVVDKFNTAYDKH-SRRGGRTAFM------------------           317
                *
```

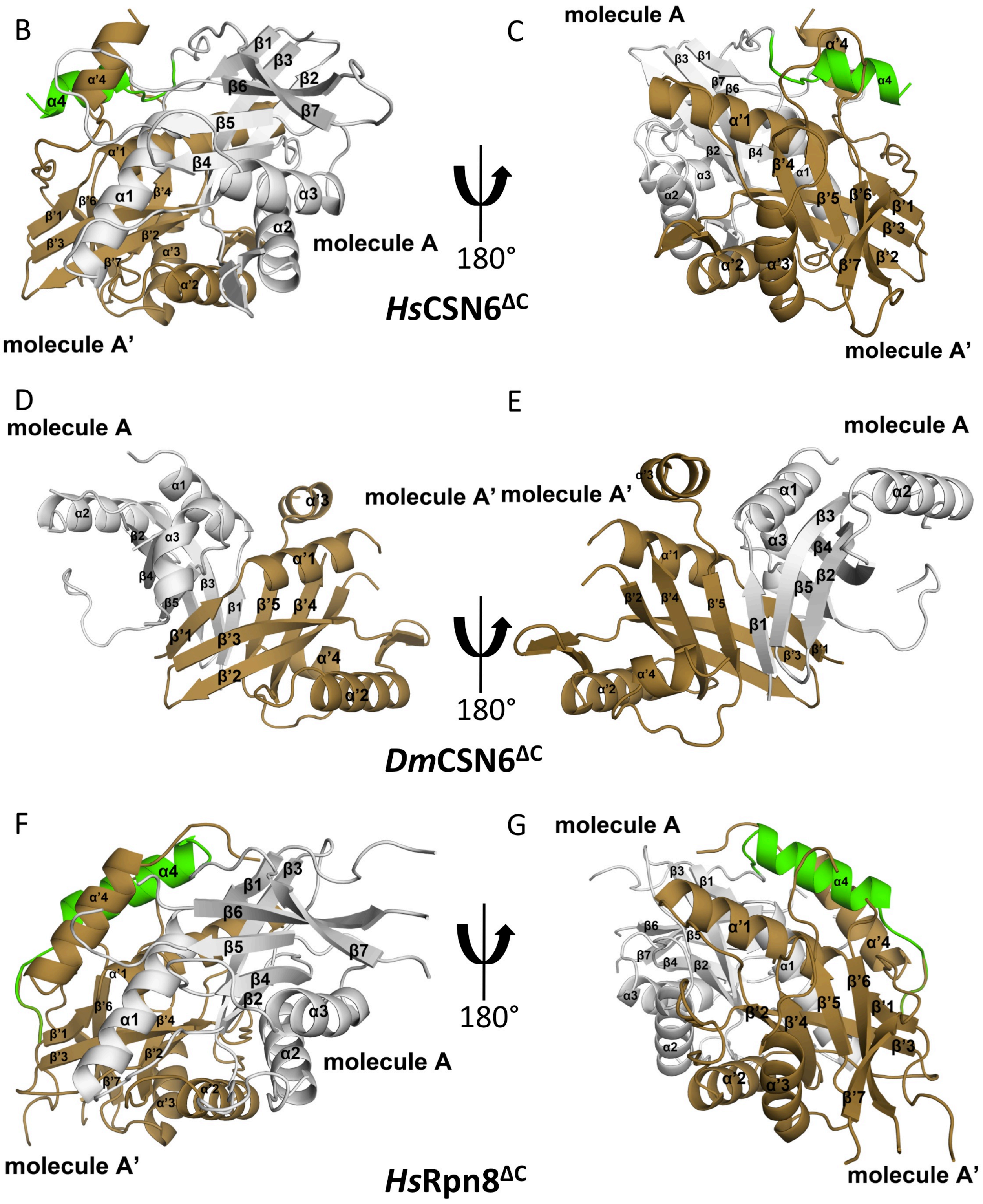

Figure S4. CSN6 sequences and MPN domain structure. (A) Sequence alignment of human CSN6 and CSN6 sequences from 10 diverse representative organisms. To obtain this sequence alignment, CSN6 sequences were retrieved with a Blastp step using human CSN6 as the search and 10 diverse sequences from representative organisms were selected in addition to the human one. A multiple sequence alignment using these eleven sequences was done with the Clustal Omega server (default parameters). This procedure was entirely carried out on the Uniprot website (www.uniprot.org). *Dm*: *Drosophila*

melanogaster (Uniprot entry: Q9VCY3); *Bm*: *Bombyx mori* (Uniprot entry: Q2F614); *Ag*: *Anopheles gambiae* (Uniprot entry: Q7Q421); *Bt*: *Bos taurus* (Uniprot entry: A6QQ21); *Mm*: *Mus musculus* (Uniprot entry: Q3UIT2); *Hs*: *Homo sapiens* (Uniprot entry: Q7L5N1); *Xl*: *Xenopus laevis* (Uniprot entry: Q6NUC2); *Dr*: *Danio rerio* (Uniprot entry: Q567F8); *Sm*: *Schistosoma mansoni* (Uniprot entry: G4V7G0); *Os*: *Oryza sativa* (Uniprot entry: Q6ZKM2); *At*: *Arabidopsis thaliana* (Uniprot entry: Q8W1P0). A '*' sign indicates a residue strictly conserved among the eleven selected sequences. The residues H44 and V115 discussed in the text are highlighted with an orange box and CSN6 residues involved in the CSN5ΔC/CSN6ΔC complex that we propose in this study are boxed in blue. **(B-G) Illustration of (B,C) human CSN6$^{\Delta C}$, (D,E) *Dm*CSN6 fragment (residues 51-187) and (F,G) human Rpn8$^{\Delta C}$ (residues 1-186) homodimers. Two orientations (0; 180°) are displayed for each protein.** The protomers in dimer are represented in brown ribbons. In human CSN6$^{\Delta C}$ and in Rpn8$^{\Delta C}$, the C-terminal helical portion (green) of molecule A is engaged in a helix swap with the molecule A'. Defined secondary structure elements are labelled. Human CSN6$^{\Delta C}$ and Rpn8$^{\Delta C}$ contain seven β-strands and four α-helices. *Dm*CSN6$^{\Delta C}$ structure contains five β-strands and two to three α-helices, depending on the considered monomer. Structural comparison of the monomeric form of *Hs*CSN6$^{\Delta C}$ with *Hs*Rpn8$^{\Delta C}$ and *Dm*CSN6$^{\Delta C}$ using LSQMAN program (2) gave rmsd values of 1.64 Å for 153 Cα pairs and 1.35 Å for 84 Cα pairs, respectively.

Reference.

2. Kleywegt, G. J. (1999) Experimental assessment of differences between related protein crystal structures. *Acta Crystallogr D Biol Crystallogr* **55**, 1878-1884
