# Supplementary material for: Structural and Biochemical Characterization of the Cop9 Signalosome CSN5/CSN6 Heterodimer
Source: PLoS One. 2014 Aug 21;9(8):e105688. doi: 10.1371/journal.pone.0105688 (PMC4140821; doi:10.1371/journal.pone.0105688)
Supplement: Figure S5 — Pro-Nedd8 processing by CSN5ΔC. (PDF) [file pone.0105688.s005.pdf]

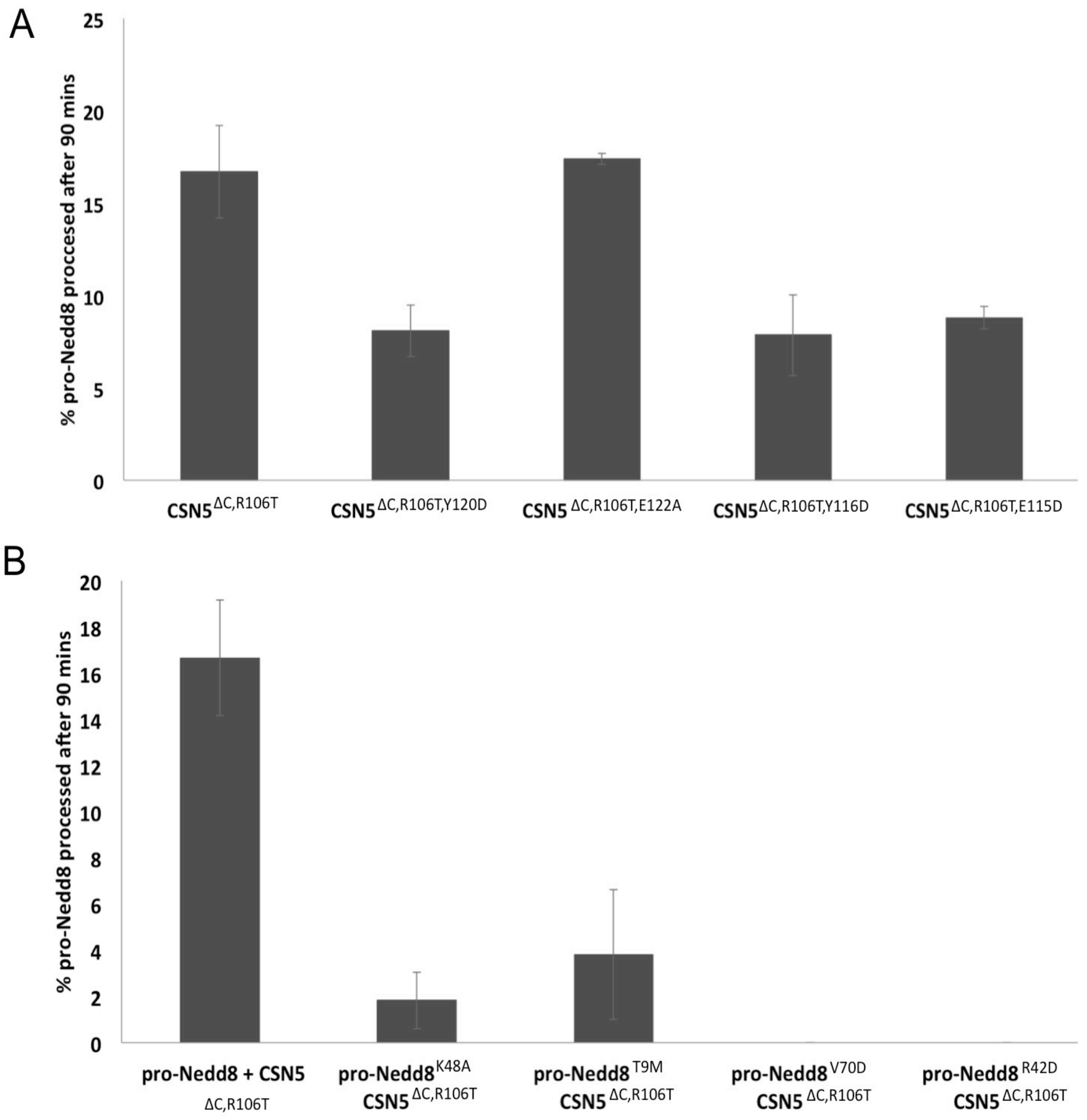

Figure S5. Pro-Nedd8 processing by CSN5$^{\Delta C}$. (A, B) The activity of CSN5$^{\Delta C,R106T}$ and variants (**A**; Y120D, E122A, Y116D, E115A) on the processing of pro-Nedd8^{WT} and variants (**B;** K48A, T9M, V70D, R42D) is displayed, as the percentage of cleaved protein after 90 min reaction. The error bar corresponds to the s.d. over two independent experiments.
