# Supplementary material for: Structural and Biochemical Characterization of the Cop9 Signalosome CSN5/CSN6 Heterodimer
Source: PLoS One. 2014 Aug 21;9(8):e105688. doi: 10.1371/journal.pone.0105688 (PMC4140821; doi:10.1371/journal.pone.0105688)
Supplement: Figure S6 — Interface residue of the CSN5ΔC/CSN6ΔC heterodimer models expressed as the number of atomic contact pairs between residues of the predicted heterodimers. (PDF) [file pone.0105688.s006.pdf]

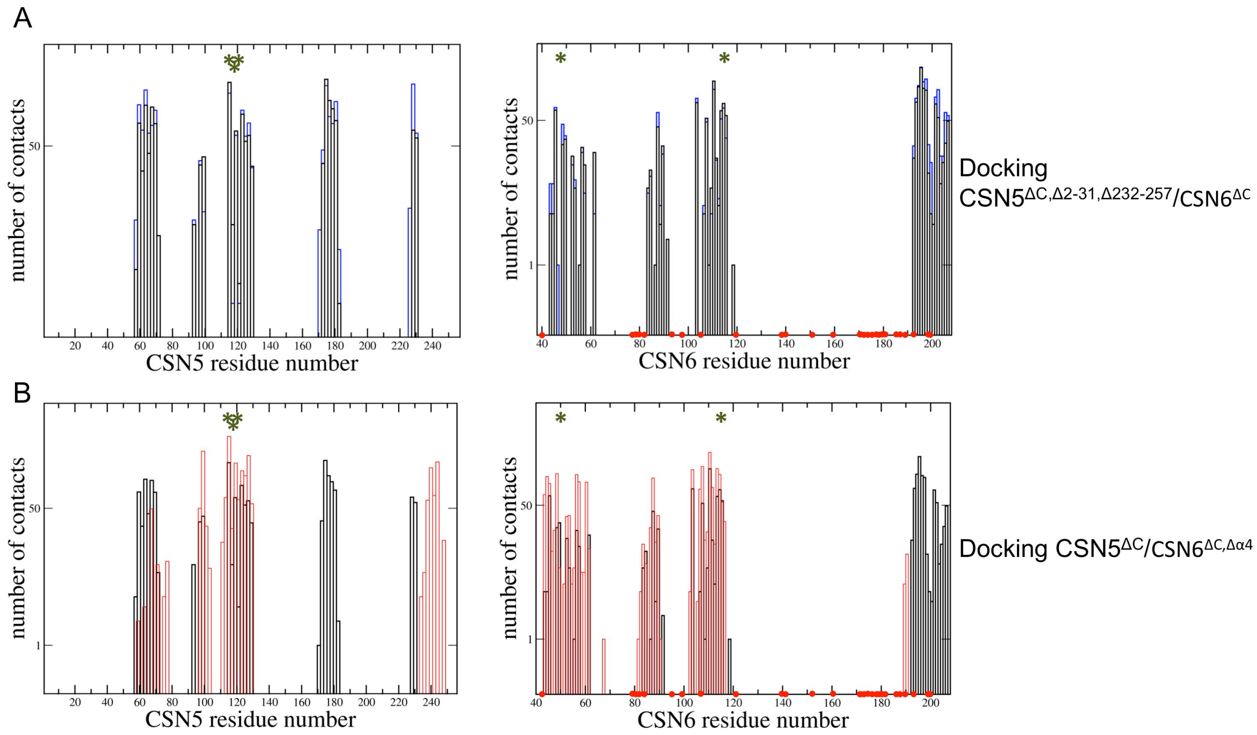


**Figure S6. Interface residue of the CSN5$^{\Delta C}$/CSN6$^{\Delta C}$ heterodimer models expressed as the number of atomic contact pairs between residues of the predicted heterodimers. (A)** CSN5$^{\Delta C,\Delta 2\text{-}31,\Delta 232\text{-}257}$ (residues 32-231) and CSN6$^{\Delta C}$ (residues 38-207). **(B)** CSN5$^{\Delta C}$ and CSN6$^{\Delta C,\Delta\alpha 4}$ (residues 38-191). The best poses obtained from filtered ZDOCK models are shown in blue and red; and the dimer superimposed on Rpn11$^{\Delta C}$/Rpn8$^{\Delta C}$ crystal structure (PDB code 4O8X) is shown in black. The buried surface area of these three complexes is 1,675 Å$^2$ (panel A-blue), 1,430 Å$^2$ (panel B-red) and 1,695 Å$^2$ (panels A and B-black). The corresponding value for the Rpn11$^{\Delta C}$/Rpn8$^{\Delta C}$ dimer (PDB code 4O8Y) is 2,006 Å$^2$. The average sequence conservation score of the residues involved in the CSN5/CSN6 (and related) interface is 52.7, 51.1 and 51.9, respectively, as compared to an average conservation score of 41.8 for all surface residues (SASA>10 Å$^2$). Residues with a green * symbol (CSN5$^{\Delta C}$ E115, Y116, Y120 and CSN6$^{\Delta C}$ H44, V115) and with a red • symbol (corresponding to the CSN6 residues that have been flagged as not perturbed by CSN5 binding by NMR HSQC; Table S4) have been used to guide the docking procedure.
