# Supplementary material for: Structural and Biochemical Characterization of the Cop9 Signalosome CSN5/CSN6 Heterodimer
Source: PLoS One. 2014 Aug 21;9(8):e105688. doi: 10.1371/journal.pone.0105688 (PMC4140821; doi:10.1371/journal.pone.0105688)
Supplement: Figure S7 — CSN5ΔC normal modes analysis using WEBnm@ server. (PDF) [file pone.0105688.s007.pdf]

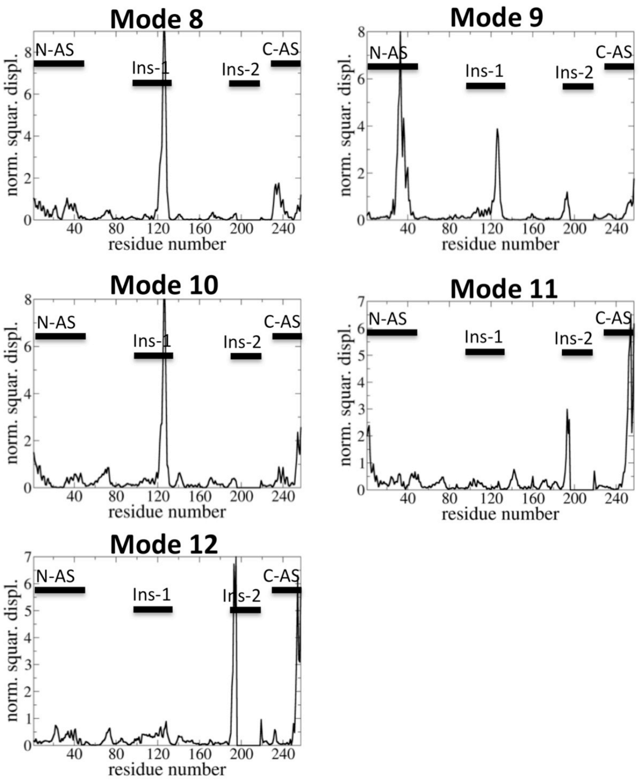

Figure S7. CSN5$^{\Delta C}$ normal modes analysis using WEBnm@ server. The normalized squared displacements for the five lowest frequency modes of CSN5$^{\Delta C}$ (PDB code 4F7O; chain B). The appending and insertion segments (AS and Ins, respectively) are displayed as bars.
