# Supplementary material for: Structural and Biochemical Characterization of the Cop9 Signalosome CSN5/CSN6 Heterodimer
Source: PLoS One. 2014 Aug 21;9(8):e105688. doi: 10.1371/journal.pone.0105688 (PMC4140821; doi:10.1371/journal.pone.0105688)
Supplement: Figure S8 — Linkage map of the CSN subunits obtained from cross-linked CSN followed by MS analysis. (PDF) [file pone.0105688.s008.pdf]

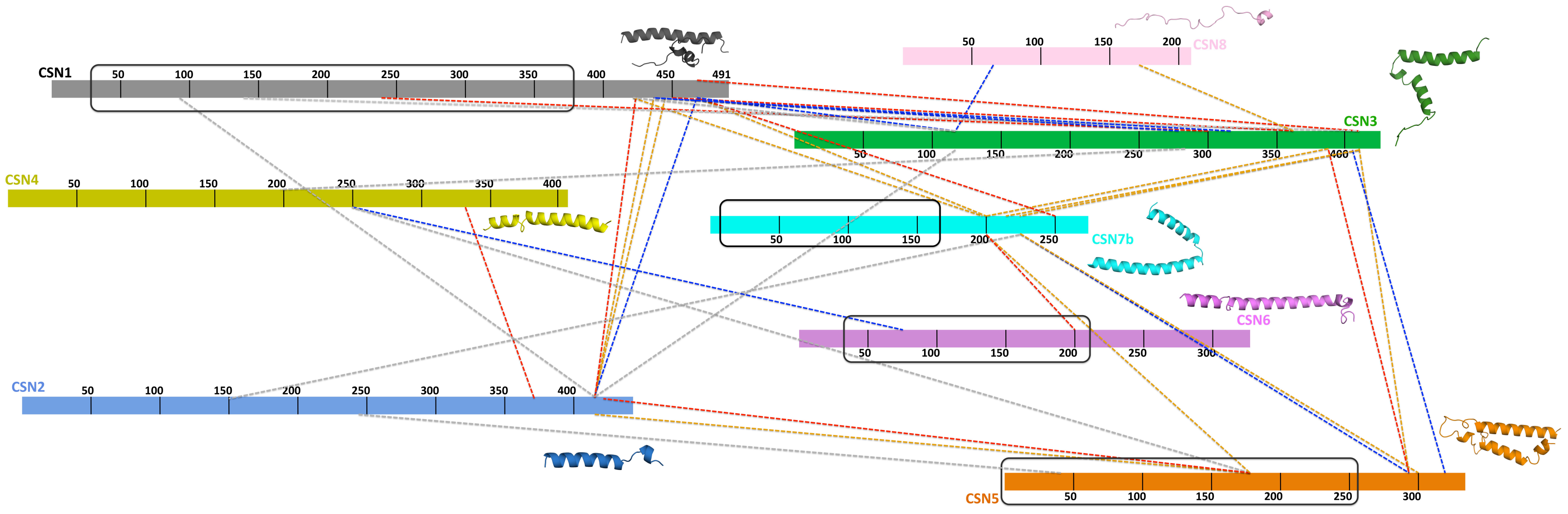

Figure S8. Linkage map of the CSN subunits obtained from cross-linked CSN followed by MS analysis. The position of the link in the sequence is displayed with dotted lines. Linkage lines are coloured coded depending on the agreement with the revisited CSN topology (red lines for Lys Cα-Cα distances < 30 Å ; orange lines for Lys Cα-Cα distances comprised between 30 and 40 Å ; blue lines for Lys Cα-Cα distances comprised between 40 and 45 Å ; grey lines for Lys Cα-Cα distances > 45 Å). Regions in rectangles correspond to the structurally characterised portions of the subunits (*Homo sapiens* CSN5 and CSN6; *Arabidopsis thaliana* CSN1 and CSN7). The modelled C-terminal helical segments contributing to the helical bundle are displayed as colored ribbons.
