# Supplementary material for: Structural and Biochemical Characterization of the Cop9 Signalosome CSN5/CSN6 Heterodimer
Source: PLoS One. 2014 Aug 21;9(8):e105688. doi: 10.1371/journal.pone.0105688 (PMC4140821; doi:10.1371/journal.pone.0105688)
Supplement: Table S1 — X ray data collection and refinement statistics for human CSN6ΔC crystal. (PDF) [file pone.0105688.s009.pdf]

Table S1. X ray data collection and refinement statistics for human CSN6$^{\Delta C}$ crystal

Data collection	CSN6$^{\Delta C}$ (residues 31-211)
PDB code	4QFT
Space group	$P3_121$
Cell dimensions (Å)	
a	96.84
b	96.84
c	48.33
Maximal resolution (Å)	1.76
Observed reflections	263,696
Unique reflections	26,172
Completeness (%)	100
R_{merge} [a]	0.104
Mean I/σ(I)	14.4
Highest resolution bin (Å)	1.86-1.76
Completeness (%)	100
R_{merge} [a]	0.492
Mean I/σ(I)	4.9
Refinement	
Protein atoms	1474
Waters	181
Resolution range (Å)	48.00-1.76
R_{conv} [b] (%)	15.5
R_{free} [c] (%)	20.6
Mean protein B factor (Å^2)	25.3
Mean solvent B factor (Å^2)	39.1
Rms deviation bond angles (°)	1.164
Rms deviation bond length (Å)	0.007
Estimated coordinate error (Å)	0.112

[a] $R_{merge} = \Sigma_h\Sigma_j |I_{h,j} - \bar{I}_h|/\Sigma_h\Sigma_j|I_{h,j}|$, where $I_{h,j}$ is the jth observation of reflection h.
[b] $R_{conv} = \Sigma_h||F_{oh}| - |F_{ch}||/\Sigma_h|F_{oh}|$, where F_{oh} and F_{ch} are the observed and calculated structure factor amplitudes respectively for the reflection h.
[c] R_{free} is equivalent to R_{conv} for a 5% subset of reflections not used in the refinement
