# Supplementary material for: Structural and Biochemical Characterization of the Cop9 Signalosome CSN5/CSN6 Heterodimer
Source: PLoS One. 2014 Aug 21;9(8):e105688. doi: 10.1371/journal.pone.0105688 (PMC4140821; doi:10.1371/journal.pone.0105688)
Supplement: Table S2 — NMR experiments acquired for chemical shift assignments. (PDF) [file pone.0105688.s010.pdf]

Table S2. NMR experiments acquired for chemical shift assignments

		Size			Sweep width (ppm)					
Experiments	nuclei	F3	F2	F1	F3	F2	F1	Mix (ms)	NS	D1(s)
^{15}N-HSQC	^{1}H, ^{15}N	1500	160	-	14	20.42	-	-	16	1
^{15}N-NOESY-HSQC	^{1}H,^{15}N, ^{1}H	1500	64	320	14	20.42	14	120	12	1
^{15}N-TOCSY-HSQC	^{1}H,^{15}N, ^{1}H	1500	48	256	14	20.42	14	46.8	12	1

Experiments were recorded using WATERGATE water suppression from Topspin (v. 2.1) pulse program library.
