# Supplementary material for: Structural and Biochemical Characterization of the Cop9 Signalosome CSN5/CSN6 Heterodimer
Source: PLoS One. 2014 Aug 21;9(8):e105688. doi: 10.1371/journal.pone.0105688 (PMC4140821; doi:10.1371/journal.pone.0105688)
Supplement: Table S3 — NMR experiments acquired for CSN5ΔC/CSN6ΔC mapping. (PDF) [file pone.0105688.s011.pdf]

Table S3. NMR experiments acquired for $CSN5^{\Delta C}$/$CSN6^{\Delta C}$ **mapping**

		Size			Sweep width (ppm)					
Experiments	nuclei	F3	F2	F1	F3	F2	F1	Mix (ms)	NS	D1(s)
^{15}N-HSQC	^{1}H, ^{15}N	1500	160	-	14	34	-	-	64	1
