# Supplementary material for: Structural and Biochemical Characterization of the Cop9 Signalosome CSN5/CSN6 Heterodimer
Source: PLoS One. 2014 Aug 21;9(8):e105688. doi: 10.1371/journal.pone.0105688 (PMC4140821; doi:10.1371/journal.pone.0105688)
Supplement: Table S4 — Unperturbed CSN6ΔC residues upon addition of CSN5ΔC as evaluated by NMR HSQC. (PDF) [file pone.0105688.s012.pdf]

Table S4: Unperturbed CSN6$^{\Delta C}$ **residues upon addition of** CSN5$^{\Delta C}$ **as evaluated by NMR HSQC.** Residues were considered as unperturbed those with Euclidian distances less than 0.077 ppm (1). Residues marked with an asterisk (*) are disordered in the human CSN5$^{\Delta C}$ crystal structure.

C33*	V171
G34*	I172
V35*	D173
T36*	I174
G37*	I175
S40	N176
G78	G177
R79	A179
N80	T180
M84	L182
L90	L186
K97	T187
E109	T189
L119	E196
H139	G199
K140	V200
F151	H202
K159	T209*

Reference.

1. Williamson, M. P. (2013) Using chemical shift perturbation to characterise ligand binding. *Prog Nucl Magn Reson Spectrosc* **73**, 1-16
