# Supplementary material for: Structural and Biochemical Characterization of the Cop9 Signalosome CSN5/CSN6 Heterodimer
Source: PLoS One. 2014 Aug 21;9(8):e105688. doi: 10.1371/journal.pone.0105688 (PMC4140821; doi:10.1371/journal.pone.0105688)
Supplement: Table S5 — Cross-links used in this study. (PDF) [file pone.0105688.s013.pdf]

Table S5. Cross-links used in this study. "**Id**" denotes the exact amino acid sequence of the cross-linked peptide and the exact position of the cross-link. E.g. AYAVGDVEKAALVK-FNIKSSHPQSPR-a9-b4 indicates that the lysine on position 9 in the first peptide was cross-linked with the lysine on position 4 in the second peptide. This is defined as a "unique cross-link". It is important to note, that multiple identifications of unique cross-links within one sample, including technical replicates, were never counted. "**Protein1**" and "**Protein2**" is the protein identifier for the cross-linked peptides. "**Residue 1**" and "**Residue 2**" defines the position of the cross-linked lysine within the sequence of the protein. "**deltaS**" gives the delta score of the respective cross-link and is a measure for how close the best assigned hit was scored in regard to the second best. "**Id_Score**" is a weighted sum of four subscores: xcorrc, xcorrx, match-odds and TIC that is used to assess the quality of the composite MS2 spectrum as calculated by *xQuest*. "**FDR**" denotes the false-discovery rate as calculated by *xProphet*. "**Xlink_type**" indicates the nature of the respective cross-link (inter- or intralink). "**Mapping**"indicates the position of the cross-link when mapped onto CSN subunits. CTH denotes C-terminal helix, WH stands for winged helix. For this study only interlinks were used for modelling.

Interlinks

Id	Protein1	Protein2	Residue 1	Residue 2	deltaS	ld-Score	FDR	XLType	Mapping
SQEGRPVQVIGALIGKQEGR-MLATLFKDER-a16-b7	Csn6	Csn4	75	251	0.43	44.68	0	inter-protein	MPN6-PCI4
AMDQEITVNPQFVQKSMGSQEDDSGNKPSSYS-NQIHVKSPPR-a15-b6	Csn3	Csn1	443	447	0.21	44.39	0	inter-protein	CTH3 - CTH1
YTALDKWTNQLNSLNQAVVSK-STTFEKSLLMGK-a6-b6	Csn2	Csn1	426	418	0.32	41.46	0	inter-protein	CTH2 - CTH1
NLVNKHSETFTR-NQIHVKSPPR-a5-b6	Csn3	Csn1	318	447	0.48	41.25	0	inter-protein	PCI3-CTH1
IPPAIKSANSELGGIWSVGQR-KQPLR-a6-b1	Csn8	Csn3	65	152	0.41	41.2	0	inter-protein	Nterm8-Nterm3
VDSHSKILYAR-ANQYKENHNR-a6-b5	Csn1	Csn7	402	199	0.49	39.73	0	inter-protein	CTH1 - CTH7
AEKIASQMITEGR-TQVLIKLIKPYTR-a3-b6	Csn4	Csn2	337	361	0.9	39.33	0	inter-protein	WH4-WH2
LKAMDQEITVNPQFVQK-ANQYKENHNR-a2-b5	Csn3	Csn7	428	199	0.31	39.17	0	inter-protein	CTH3-CTH7
TQQQVEAEVTNIKK-KSEDKLAK-a13-b1	Csn7	Csn5	217	295	0.7	36.81	0	inter-protein	CTH7-CTH5
TISAGKVNLGAFR-ANQYKENHNR-a6-b5	Csn5	Csn7	180	199	0.88	36.58	0	inter-protein	MPN5-CTH7
NLVNKHSETFTR-KLSEATR-a5-b1	Csn3	Csn1	318	81	0.57	36.25	0	inter-protein	PCI3 - Nterm1
NLVNKHSETFTR-VDSHSKILYAR-a5-b6	Csn3	Csn1	318	402	0.62	35.83	0	inter-protein	PCI3-CTH1
NQIHVKSPPREGSQGELTPANSQSR-IDQVNQLLELDHQKR-a6-b14	Csn1	Csn2	447	415	0.63	35.45	0	inter-protein	CTH1-CTH2
AMDQEITVNPQFVQKSMGSQEDDSGNKPSSYS-DKLFNQINIS-a15-b2	Csn3	Csn5	443	326	0.91	35.28	0	inter-protein	
IDQVNQLLELDHQKR-NQIHVKSPPR-a14-b6	Csn2	Csn1	415	447	0.64	34.67	0.036	inter-protein	WH domain/CTH2-CTH1
LTKTFLTLSLQDMASR-STTFEKSLLMGKEFQR-a3-b6	Csn3	Csn1	349	418	0.25	34.35	0.036	inter-protein	WH domain3 - CTH1
NQIHVKSPPREGSQGELTPANSQSR-SMGSQEDDSGNKPSSYS-a6-b12	Csn1	Csn3	447	455	0.21	34.22	0.036	inter-protein	CTH1-CTH3
YTALDKWTNQLNSLNQAVVSK-STTFEKSLLMGKEFQR-a6-b6	Csn2	Csn1	426	418	0.93	33.97	0.036	inter-protein	CTH2 - CTH1
SMGSQEDDSGNKPSSYS-NQIHVKSPPR-a12-b6	Csn3	Csn1	455	447	0	33.76	0.036	inter-protein	CTH3-CTH1
AMDQEITVNPQFVQKSMGSQEDDSGNKPSSYS-NQIHVKSPPR-a27-b6	Csn3	Csn1	455	447	0.28	33.59	0.036	inter-protein	CTH3-CTH1
IDQVNQLLELDHQKR-VDSHSKILYAR-a14-b6	Csn2	Csn1	415	402	0.45	33.58	0.036	inter-protein	WH domain/CTH2-CTH1
IDQVNQLLELDHQKR-TISAGKVNLGAFR-a14-b6	Csn2	Csn5	415	180	0.5	33.47	0.036	inter-protein	CTH2-MPN5
TISAGKVNLGAFR-MLATLFKDER-a6-b7	Csn5	Csn4	180	251	0.35	33.41	0.036	inter-protein	MPN5-PCI4
TQQQVEAEVTNIKK-KSEDKLAK-a13-b5	Csn7	Csn5	217	299	0.66	33.37	0.036	inter-protein	CTH7-CTH5
NLVNKHSETFTR-KFIEAAQR-a5-b1	Csn3	Csn4	318	200	0.3	32.87	0.036	inter-protein	PCI3-Nterm4
SMGSQEDDSGNKPSSYS-KSEDKLAK-a12-b1	Csn3	Csn5	455	295	0	32.07	0.036	inter-protein	CTH3-CTH5
LTKTFLTLSLQDMASR-STTFEKSLLMGK-a3-b6	Csn3	Csn1	349	418	0.18	31.99	0.036	inter-protein	WH3-CTH1
TLKATASSSAQEMEQQLAER-LGKLYLEREEYGK-a3-b3	Csn7	Csn2	221	157	0.94	31.85	0.036	inter-protein	CTH7-Nterm2
YTALDKWTNQLNSLNQAVVSK-TISAGKVNLGAFR-a6-b6	Csn2	Csn5	426	180	0.66	31.69	0.062	inter-protein	CTH2-MPN5
NQIHVKSPPREGSQGELTPANSQSR-KQPLR-a6-b1	Csn1	Csn3	447	152	0.54	31.51	0.062	inter-protein	CTH1-Nterm3
LKAMDQEITVNPQFVQK-KSEDK-a2-b1	Csn3	Csn5	428	295	0.34	31.41	0.062	inter-protein	CTH3-CTH5
TQQQVEAEVTNIKK-KSEDK-a13-b1	Csn7	Csn5	217	295	0.29	31.35	0.062	inter-protein	CTH7-CTH5
SMGSQEDDSGNKPSSYS-KSEDKLAK-a12-b5	Csn3	Csn5	455	299	0.28	30.19	0.083	inter-protein	CTH3-CTH5
VDSHSKILYAR-KQPLR-a6-b1	Csn1	Csn3	402	152	0.53	30.18	0.083	inter-protein	CTH1-Nterm3
DGMVSFHDNPEKYNNPAMLHNIDQEMLK-KPVAGALDVSFNK-a12-b1	Csn3	Csn8	403	166	0.23	29.84	0.083	inter-protein	CTH3-CTH8
IDQVNQLLELDHQKR-KQPLR-a14-b1	Csn2	Csn3	415	152	0.63	29.74	0.083	inter-protein	CTH2-Nterm3
DNNMGLVKQCLSSLYK-KYKQAAK-a8-b3	Csn3	Csn1	333	244	0.92	29.26	0.108	inter-protein	PCI3-PCI1
IDQVNQLLELDHQKR-KLSEATR-a14-b1	Csn2	Csn1	415	81	0.89	29.01	0.116	inter-protein	CTH2-Nterm1
NQIHVKSPPREGSQGELTPANSQSR-QPTKK-a6-b4	Csn1	Csn7	447	251	0.53	28.99	0.116	inter-protein	CTH1-CTH7
IDQVNQLLELDHQKR-STTFEKSLLMGK-a14-b6	Csn2	Csn1	415	418	0.54	28.6	0.116	inter-protein	CTH2-CTH1
SMGSQEDDSGNKPSSYS-TQQQVEAEVTNIKK-a12-b13	Csn3	Csn7	455	217	0.14	28.49	0.116	inter-protein	CTH3-CTH7
DGMVSFHDNPEKYNNPAMLHNIDQEMLK-SLLMGKEFQR-a12-b6	Csn3	Csn1	403	424	0.19	28.37	0.116	inter-protein	CTH3-CTH1

NQIHVKSPPREGSQGELTPANSQSR-ANQYKENHNR-a6-b5	Csn1	Csn7	447	199	0.64	27.33	0.156	inter-protein	CTH1-CTH7
MTATGSGENSTVAEHLIAQHSAIKMLHSR-ANQYKENHNR-a24-b5	Csn6	Csn7	229	199	0.93	27.21	0.156	inter-protein	not modelled
TLKATASSSAQEMEQQLAER-SMGSQEDDSGNKPSSYS-a3-b12	Csn7	Csn3	221	455	0	26.8	0.17	inter-protein	CTH7-CTH3
QQQEILAAKPWTK-ECGGKMHLR-a9-b5	Csn5	Csn2	43	243	0.45	25.92	0.188	inter-protein	Nterm5-PCI2
SMGSQEDDSGNKPSSYS-GNSIKESIR-a12-b5	Csn3	Csn1	455	138	0	25.2	0.2	inter-protein	CTH3-Cterm1

Intralinks

Id	Protein1	Protein2	Residue 1	Residue 2	deltaS	ld-Score	FDR	XLType
VDSHSKILYAR-AKAMMLR-a6-b2	Csn1	Csn1	402	431	0.62	50.75	0	intra-protein xl
LGKLYLEREEYGK-NDRLWFKTNTK-a3-b7	Csn2	Csn2	157	150	0.61	49.8	0	intra-protein xl
LEKLDTDLKNYK-KKALLK-a3-b2	Csn1	Csn1	124	117	0.49	49.62	0	intra-protein xl
VDSHSKILYAR-RAKAMMLR-a6-b3	Csn1	Csn1	402	431	0.41	48.37	0	intra-protein xl
NQIHVKSPPR-AKAMMLR-a6-b2	Csn1	Csn1	447	431	0.52	43.11	0	intra-protein xl
SLLMGKEFQR-RAKAMMLR-a6-b3	Csn1	Csn1	424	431	0.91	42.79	0	intra-protein xl
SLLMGKEFQR-AKAMMLR-a6-b2	Csn1	Csn1	424	431	0.87	42.26	0	intra-protein xl
LEKLDTDLKNYK-KALLK-a3-b1	Csn1	Csn1	124	117	0.54	41.97	0	intra-protein xl
TLKATASSSAQEMEQQLAER-TQQQVEAEVTNIKK-a3-b13	Csn7	Csn7	221	217	0.44	41.78	0	intra-protein xl
VDSHSKILYAR-NQIHVKSPPR-a6-b6	Csn1	Csn1	402	447	0.54	41.54	0	intra-protein xl
IDQVNQLLELDHQKR-LIKPYTR-a14-b3	Csn2	Csn2	415	364	0.69	41.25	0	intra-protein xl
LTKTFLTLSLQDMASR-KNIQR-a3-b1	Csn3	Csn3	349	342	0.48	40.93	0	intra-protein xl
VLELEGEKGEWGFK-YKQLLTYIR-a8-b2	Csn2	Csn2	64	93	0.22	40.81	0	intra-protein xl
ALKEDDPKAALSSFQK-INFKLTNFPEMMNR-a8-b4	Csn2	Csn2	48	81	0.26	40.11	0	intra-protein xl
NDRLWFKTNTK-LGKLYLER-a7-b3	Csn2	Csn2	150	157	0.45	40.11	0	intra-protein xl
SLLMGKEFQRR-AKAMMLR-a6-b2	Csn1	Csn1	424	431	0.94	39.2	0	intra-protein xl
LEKLDTDLK-KALLK-a3-b1	Csn1	Csn1	124	117	0.49	39.19	0	intra-protein xl
GIGILKQAIDK-KQPLR-a6-b1	Csn3	Csn3	162	152	0.85	37.62	0	intra-protein xl
FIKPLSNAYHELAQVYSTNNPSELR-VQQLPKYTSQIVGR-a3-b6	Csn3	Csn3	291	280	0.82	37.38	0	intra-protein xl
LGKLYLER-LWFKTNTK-a3-b4	Csn2	Csn2	157	150	0.39	37.32	0	intra-protein xl
LEKLDTDLK-KKALLK-a3-b2	Csn1	Csn1	124	117	0.59	37.15	0	intra-protein xl
NQIHVKSPPREGSQGELTPANSQSR-VDSHSKILYAR-a6-b6	Csn1	Csn1	447	402	0.42	37.11	0	intra-protein xl
IPPAIKSANSELGGIWSVGQR-YLWKR-a6-b4	Csn8	Csn8	65	58	0.48	36.48	0	intra-protein xl
VDSHSKILYAR-KLSEATR-a6-b1	Csn1	Csn1	402	81	0.7	35.98	0	intra-protein xl
NQIHVKSPPR-RAKAMMLR-a6-b3	Csn1	Csn1	447	431	0.67	35.52	0	intra-protein xl
NLVNKHSETFTR-KQPLR-a5-b1	Csn3	Csn3	318	152	0.39	34.95	0.061	intra-protein xl
GNQLQEFAAMLMPHQKATTADGSSILDR-KFIEAAQR-a16-b1	Csn4	Csn4	290	200	0.39	34.8	0.061	intra-protein xl
STTFEKSLLMGK-VDSHSKILYAR-a6-b6	Csn1	Csn1	418	402	0.41	34.64	0.061	intra-protein xl
ALKEDDPKAALSSFQK-INFKLTNFPEMMNR-a3-b4	Csn2	Csn2	43	81	0.27	34.23	0.061	intra-protein xl
GSFMLGLETHDRKSEDK-LAKATR-a13-b3	Csn5	Csn5	295	302	0.48	34.21	0.061	intra-protein xl
NQIHVKSPPREGSQGELTPANSQSR-AKAMMLR-a6-b2	Csn1	Csn1	447	431	0.38	33.86	0.061	intra-protein xl
LGKLYLEREEYGK-LWFKTNTK-a3-b4	Csn2	Csn2	157	150	0.68	33.37	0.061	intra-protein xl
NQIHVKSPPREGSQGELTPANSQSR-RAKAMMLR-a6-b3	Csn1	Csn1	447	431	0.47	33.37	0.061	intra-protein xl
LEKLDTDLK-KKALLK-a3-b1	Csn1	Csn1	124	116	0.76	33.06	0.083	intra-protein xl
NLVNKHSETFTR-NLVNKHSETFTR-a5-b5	Csn3	Csn3	318	318	0.53	32.79	0.083	intra-protein xl
LEKLDTDLK-NYKGNSIK-a3-b3	Csn1	Csn1	124	133	0.76	32.18	0.083	intra-protein xl
MTATGSGENSTVAEHLIAQHSAIKMLHSR-VKLILEYVK-a24-b2	Csn6	Csn6	229	236	0.6	31.95	0.105	intra-protein xl

DGMVSFHDNPEKYNNPAMLHNIDQEMLK-LTKTFLTLSLQDMASR-a12-b3	Csn3	Csn3	403	349	0.84	30.85	0.105	intra-protein xl
QHLASIYEKEEDWR-KFIEAAQR-a9-b1	Csn4	Csn4	116	200	0.54	30.65	0.111	intra-protein xl
FIKPLSNAYHELAQVYSTNNPSELR-NLVNKHSETFTR-a3-b5	Csn3	Csn3	291	318	0.32	30.55	0.111	intra-protein xl
YTALDKWTNQLNSLNQAVVSK-IDQVNQLLELDHQKR-a6-b14	Csn2	Csn2	426	415	0.48	30.32	0.111	intra-protein xl
GNSIKESIRR-LDTDLKNYK-a5-b6	Csn1	Csn1	138	130	0.56	30	0.111	intra-protein xl
TISAGKVNLGAFR-KSEDKLAK-a6-b5	Csn5	Csn5	180	299	0.59	29.99	0.111	intra-protein xl
EGEFEKAHTDFFEAFKNYDESGSPR-ALYEQSLHIKSAIPHPLIMGVIR-a6-b10	Csn2	Csn2	253	225	0.79	29.6	0.111	intra-protein xl
NYSEKSINSILDYISTSK-NDRLWFKTNTK-a5-b7	Csn2	Csn2	110	150	0.91	29	0.111	intra-protein xl
TQQQVEAEVTNIKK-ANQYKENHNR-a13-b5	Csn7	Csn7	217	199	0.89	27.81	0.128	intra-protein xl
ALYEQSLHIKSAIPHPLIMGVIR-EGEFEKAHTDFFEAFK-a10-b6	Csn2	Csn2	225	253	0.43	26.81	0.128	intra-protein xl
NQIHVKSPPREGSQGELTPANSQSR-KLSEATR-a6-b1	Csn1	Csn1	447	81	0	26.43	0.16	intra-protein xl
STTFEKSLLMGK-AKAMMLR-a6-b2	Csn1	Csn1	418	431	0.55	26.13	0.16	intra-protein xl
AMDQEITVNPQFVQKSMGSQEDDSGNKPSSYS-NLVNKHSETFTR-a15-b5	Csn3	Csn3	443	318	0.7	26.09	0.16	intra-protein xl
