# Supplementary material for: Structural and Biochemical Characterization of the Cop9 Signalosome CSN5/CSN6 Heterodimer
Source: PLoS One. 2014 Aug 21;9(8):e105688. doi: 10.1371/journal.pone.0105688 (PMC4140821; doi:10.1371/journal.pone.0105688)
Supplement: Table S6 — Sequences of the CSN subunit models used. (PDF) [file pone.0105688.s014.pdf]

Table S6. Sequences of the CSN subunit models used. The Phyre models for CSN1 and CSN7 are based on the crystal structures of their respective *Arabidopsis thaliana* homologs (PDB codes 4LCT and 3CHM, respectively).

Protein	Sequence	Boundaries	Model
**Human CSN1**	SLDLEQYAASYSGLMRIERLQFIADHCPTLRVEALKMALSFVQRTFNVDMYEEIHRKLSEATREPPALDTAWVEAT<br>RKKALLKLEKLDTDLKNYKGNSIKESIRRGHDDLGDHYLDCGDLSNALKCYSRARDYCTSAKHVINMCLNVIKVSVY<br>LQNWSHVLSYVSKAESTPEIAEQRGERDSQTQAILTKLKCAAGLAELAARKYKQAAKCLLLASFDHCDFPELLSPS<br>NVAIYGGLCALATFDRQELQRNVISSSSFKLFLELEPQVRDIIFKFYESKYASCLKMLDEMKDNLLLDMYLAPHVRTL<br>YTQIRNRALIQYFSPYVSADMHRMAAAFNTTVAALEDELTQLILEGLISARVDSHSKILYARDVDQRSTTFEKSLLMG<br>KEFQRRAKAMMLRAAVLRNQIHVKSPPREGSQGELTPANSQSRMSTNM	41-491<br>Δ104-121	Phyre model
**Human CSN2**	MSDMEDDFMCDDEEDYDLEYSEDSNSEPNVDLENQYYNSKALKEDDPKAALSSFQKVLELEGEKGEWGFKALKQ<br>MIKINFKLTNFPEMMNRYKQLLTYIRSAVTRNYSEKSINSILDYISTSKQMDLLQEFYETTLEALKDAKNDRLWFKTN<br>TKLGKLYLEREEYGKLQKILRQLHQSCQTDDGEDDLKKGTQLLEIYALEIQMYTAQKNNKKLKALYEQSLHIKSAIPH<br>PLIMGVIRECGGKMHLREGEFEKAHTDFFEAFKNYDESGSPRRTTCLKYLVLANMLMKSGINPFDSQEAKPYKNDP<br>EILAMTNLVSAYQNNDITEFEKILKTNHSNIMDDPFIREHIEELLRNIRTQVLIKLIKPYTRIHIPFISKELNIDVADVESLL<br>VQCILDNTIHGRIDQVNQLLELDHQKRGGARYTALDKWTNQLNSLNQAVVSKLA	1-443	Phyre model
**Human CSN3**	MASALEQFVNSVRQLSAQGQMTQLCELINKSGELLAKNLSHLDTVLGALDVQEHSLGVLAVLFVKFSMPSVPDFET<br>LFSQVQLFISTCNGEHIRYATDTFAGLCHQLTNALVERKQPLRGIGILKQAIDKMQMNTNQLTSIHADLCQLCLLAKC<br>FKPALPYLDVDMMDICKENGAYDAKHFLCYYYYGGMIYTGLKNFERALYFYEQAITTPAMAVSHIMLESYKKYILVS<br>LILLGKVQQLPKYTSQIVGRFIKPLSNAYHELAQVYSTNNPSELRNLVNKNNMGLVKQCLSSLYKKNIQRLTKTFLTL<br>SLQDMASRVQLSGPQEAEKYVLHMIEDGEIFASINQKDEKYNNPAMLHNIDQEMLKCIELDERLKAMDQEITVNPQ<br>FVQKSMGSQEDDSGNKPSSYS	1-423<br>Δ282-289<br>Δ356-364	Phyre model
**Human CSN4**	MAAAVRQDLAQLMNSSGSHKDLAGKYRQILEKAIQLSGAEQLEALKAFVEAMVNENVSLVISRQLLTDFCTHLPNL<br>PDSTAKEIYHFTLEKIQPRVISFEEQVASIRQHLASIYEKEEDWRNAAQVLVGIPLETGQKQYNVDYKLETYLKIARLY<br>LEDDDPVQAEAYINRASLLQNESTNEQLQIHYKVCYARVLDYRRKFIEAAQRYNELSYKTIVHESERLEALKHALHC<br>TILASAGQQRSRMLATLFKDERCQQLAAYGILEKMYLDRIIRGNQLQEFAAMLMPHQKATTADGSSILDRAVIEHNL<br>LSASKLYNNITFEELGALLEIPAAKAEKIASQMITEGRMNGFIDQIDGIVHFETREALPTWDKQIQSLCFQVNNLLEKIS<br>QTAPEWTAQAMEAQMAQ	1-406	Phyre model
**Human CSN5**	AASGSGMAQKTWELANNMQEAQSIDEIYKYDKKQQQEILAAKPWTKDHHYFKYCKISALALLKMVMHARSGGNLE<br>VMGLMLGKVDGETMIIMDSFALPVEGTETRVNAQAAAYEYMAAYIENAKQVGRLENAIGWYHSHPGYGCWLSGID<br>VSTQMLNQQFQEPFVAVVIDPTRTISAGKVNLGAFRTYPKGYKPPDKQYYALEVSYFKSSSSLLTNADYTTGQVFD<br>LSEKLEQSEAQLGRGSFMLGLETHDRKSEDKLAKATRDSCKTTIEAIHGLMSQVIKDKLFN	2-229<br>Δ198-218<br>Δ232-251	X-ray structure + Phyre model
**Human CSN6**	SVSVALHPLVILNISDHWIRMRSQEGRPVQVIGALIGKQEGRNIEVMNSFELLSHTVEEKIIDKEYYYTKEEQFKQVF<br>KELEFLGWYTTGGPPDPSDIHVHKQVCEIIESPLFLKLNPMTKHTDLPVSVFESVIDIINGEATMLFAELTYTLATEEA<br>ERIGVDHVARMTAHLIAQHSAIKMLHSRVKLILEYVKASEAGEVPFNHEILREAYALCHCLPVLSTDKFKTDFYDQCN<br>DVGLMAYLGTITKTCNTMNQFVNKFNVLYDRQGIGRRMR	38-323<br>Δ209-219	X-ray structure + Phyre model
**Human CSN7b**	MAGEQKPSSNLLEQFILLAKGTSGSALTALISQVLEAPGVYVFGELLELANVQELAEGANAAYLQLLNLFAYGTYPD<br>YIANKESLPELSTAQQNKLKHLTIVSLASRMKCIPYSVLLKDLEMRNLRELEDLIIEAVYTDIIQGKLDQRNQLLEVDFC<br>IGRDIRKKDINNIVKTLHEWCDGCEAVLLGIEQQVLRANQYKENHNRTQQQVEAEVTNIKKTLKATASSSAQEMEQ<br>QLAERECPPHAEQRQPTKKMSKVKGLVSSRH	1-264	Phyre model
**Human CSN8**	MAESAFSFKKLLDQCENQELEAPGGIATPPVYGQLLALYLLHNDMNNARYLWKRI<br>PPAIKSANSELGGIWSVGQRIWQRDFPGIYTTINAHQWSETVQPIMEALRDATRRR<br>AFALVSQAYTSIIADDFAAFVGLPVEEAVKGILEQGWQADSTTRMVLPRKPVAGAL<br>DVSFNKFIPQQLARL	6-200<br>Δ182-194	Phyre model
