# Supplementary material for: Structural and Biochemical Characterization of the Cop9 Signalosome CSN5/CSN6 Heterodimer
Source: PLoS One. 2014 Aug 21;9(8):e105688. doi: 10.1371/journal.pone.0105688 (PMC4140821; doi:10.1371/journal.pone.0105688)
Supplement: Figure S2 — SDS-Page of CSN5ΔC and CSN6ΔC proteins. (PDF) [file pone.0105688.s002.pdf]

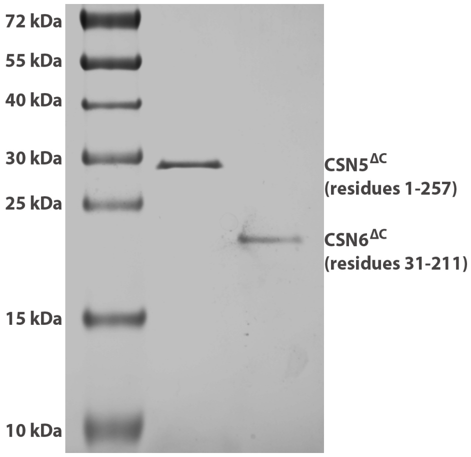

Figure S2. SDS-Page of CSN5$^{\Delta C}$ and CSN6$^{\Delta C}$ proteins. Molecular weight markers are shown to the left of the gel.
